# Recent Progress in In-Ear EEG Technology and Its Emerging Real-World Applications: A Review

**DOI:** 10.3390/mi17070764

**Published:** 2026-06-23

**Authors:** Haoqing Yan, Xin Xu

**Affiliations:** 1 School of Chemistry and Life Sciences, Nanjing University of Posts and Telecommunications, Nanjing 210023, China; hqyannj@163.com; 2 School of Communication and Information Engineering, Nanjing University of Posts and Telecommunications, Nanjing 210023, China

**Keywords:** Electroencephalography (EEG), in-ear EEG, electrodes, portable medical devices, EEG signal monitoring

## Abstract

Electroencephalography (EEG) is a core technique for brain activity monitoring. However, conventional EEG systems suffer from complicated setup and poor portability, which drives the development of ear EEG technology. Ear EEG is divided into in-ear and around-ear types, both with unique application strengths. This review mainly discusses in-ear EEG, as it features a compact structure and fits well with daily wearable use cases. Current research on in-ear EEG is limited to feasibility verification and small-sample experiments. Researchers have not yet combined personalized design with signal processing algorithms systematically, and multi-center clinical trials are still absent. These issues have become the major bottleneck hindering its clinical transformation. This paper reviews the latest advances in ear-EEG systems, focusing on structural innovation and material development to summarize key achievements in hardware design. It also summarizes its typical applications in brain-computer interfaces (BCI), covering steady-state responses, event-related potentials and motor imagery. Meanwhile, it analyzes the application of in-ear EEG in brain state monitoring, including sleep tracking, epilepsy detection, drowsiness evaluation and emotion recognition. Finally, future directions for in-ear EEG are outlined, including personalized design and intelligent signal processing. This review provides a technical framework for beginners and identifies key directions for future research.

## 1. Introduction

### 1.1. Background and Research Overview

Electroencephalography (EEG) represents the most widely used and effective method for recording the electrical activity of the brain, which is typically achieved by placing electrodes on the scalp surface. Conventional scalp EEG systems commonly employ 19 to 64 electrodes, while high-density configurations can extend to 128 or 256 channels. However, these systems are notably complex, require prolonged setup by trained professionals for precise electrode placement and cable connection, and strictly necessitate that subjects maintain relatively fixed postures, thereby significantly limiting physical mobility and daily usability. Against this background, ear electroencephalography (ear-EEG) has emerged as a novel and promising technique for acquiring brain signals by placing electrodes in or around the auricular region. Technically, ear EEG falls into two categories: in-ear EEG with electrodes placed in the ear canal or cavum conchae, and around-ear EEG with electrodes attached around the pinna (usually below the hairline) [1]. Notably, this review focuses on and systematically discusses in-ear EEG, which features a minimally invasive, intra-aural configuration. In this design, electrodes are fully embedded inside ear-mounted devices. Users can put on the devices directly without professional guidance. Additionally, in-ear EEG features excellent portability, wearing comfort and adaptability for long-term monitoring [2].

In clinical scenarios such as outpatient screening and long-term ambulatory monitoring, in-ear EEG can complement and partially replace traditional scalp EEG, especially for continuous neural monitoring in daily environments [3,4,5]. Its combination with BCI, cognitive monitoring and clinical neurodiagnostics also promotes the development of neurophysiological assessment [6,7,8]. Nevertheless, it still has a low signal-to-noise ratio (SNR), severe signal attenuation, a limited number of electrode channels, low spatial resolution, and poor source localization capability, accompanied by high contact impedance and unstable electrode–skin contact [9]. To address these technical limitations, researchers have put forward corresponding solutions, including low-impedance biocompatible flexible materials, AI algorithms, and standardized data analysis frameworks for clinical monitoring scenarios such as sleep assessment and epilepsy detection [10,11,12]. With further targeted optimization, in-ear EEG is expected to develop into a practical and reliable wearable system for long-term daily health monitoring.

Unlike prior reviews that mainly focus on device design, sensor architectures and working principles, this paper comprehensively summarizes the technical features, clinical applications and major challenges of in-ear EEG. Its core goal is to bridge the gap between laboratory research and real-world implementation. This review also puts forward future research directions, including personalized adaptive design and AI-driven signal processing for next-generation in-ear EEG systems.

### 1.2. Literature Search and Selection Criteria

The inclusion criteria were defined as follows. We selected literature published from 2011, when Looney first proposed the concept of in-ear EEG, to 27 February 2026. Only peer-reviewed original research articles and authoritative systematic reviews from indexed academic journals and high-level IEEE conference proceedings were included. Selected studies focused on in-ear EEG covering electrode fabrication, earpiece design, signal processing, BCI development and brain activity monitoring, with available quantitative experimental data. Literature was excluded if it met any of the following conditions: (1) studies on scalp or around-ear EEG without experimental verification of in-ear electrodes; (2) papers lacking complete experimental design and quantitative data; (3) duplicate publications from the same research group using identical datasets; (4) non-peer-reviewed grey literature such as commercial brochures, unverified preprints and incomplete dissertation abstracts.

We conducted a comprehensive search of electronic databases, including Web of Science, IEEE Xplore, ScienceDirect and PubMed, using a combination of the following keywords as our search terms: “in-ear EEG”, “intra-aural EEG”, “ear EEG” “, “ear-canal electroencephalography”, “ear-EEG in-ear”, “ear-based EEG” and “electrode”. Additional studies were identified through manual searches of reference lists from relevant articles that met our inclusion criteria. A total of 130 relevant articles were identified in our comprehensive search for research on ear-EEG technology. We extracted relevant data from each included study, including the study design, sample size, ear-EEG device used, electrode placement, data processing and analysis methods, and its applications. All eligible papers were subsequently allocated into four thematic groups covering hardware development, signal processing, practical applications, research limitations and future directions based on their core content for subsequent narrative synthesis.

## 2. Initial Development and Fundamental Principles of In-Ear EEG

**Figure 1:**
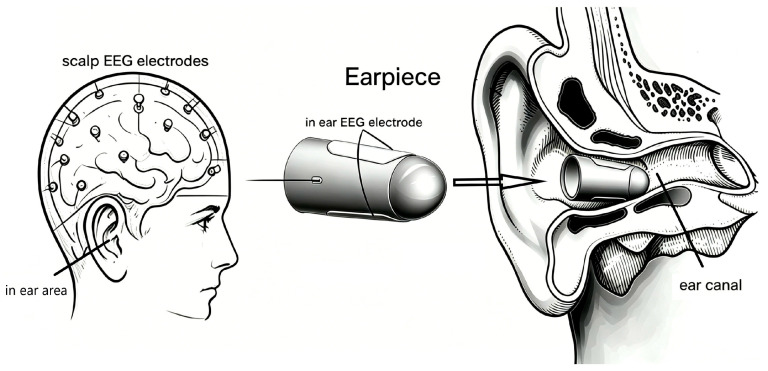
In-ear EEG device with electrodes embedded in the ear canal. (taken from [13]).

**Figure 2:**
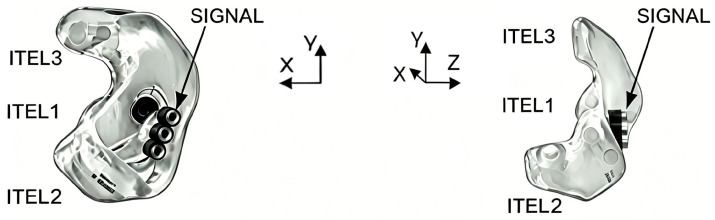
Left-ear in-ear EEG system (ITEL). Multiple electrodes (ITEL1–3) are placed on a customized earplug with distinct orientations in three projection planes. Adapted from [13].

The concept of in-ear EEG was first pioneered by Looney et al., who innovatively proposed placing electrodes within a customized earmold (Figure 1) [13]. The in-the-ear (ITE) device integrates three silver/silver chloride (AgCl) electrodes (ITEL1–ITEL3) arranged on different anatomical planes, ensuring stable and optimal contact with the ear canal wall (Figure 2) [13]. This earmold-based system delivers exceptional wearing comfort and ease of use, making it suitable for both adult and pediatric populations. Meanwhile, its reliable electrode positioning greatly improves experimental repeatability while supporting long-term continuous brain monitoring during daily activities [2]. As the widely recognized gold standard in biopotential recording, AgCl electrodes are securely embedded into these anatomically fitted earplugs to guarantee consistent and high-quality signal capture.

In-ear EEG represents a promising, non-invasive, unobtrusive, and highly practical solution for reliable and continuous brain monitoring in naturalistic daily environments. To further verify the validity of the in-ear EEG strategy, Looney et al. conducted simultaneous comparative recordings using both in-ear electrodes (Widex A/S, Lynge, Denmark) and conventional scalp electrodes [1]. Although the signals acquired by in-ear electrodes exhibited relatively lower amplitude, they benefited from reduced environmental and motion-induced noise, thus achieving a SNR comparable to that of conventional scalp EEG. As the pioneering work that formally introduced this concept, this study systematically validated the feasibility of electroencephalographic recordings through ear canal-inserted electrodes. Notably, the findings demonstrated a strong and consistent correlation between intra-auricular signals and conventional scalp signals, thereby establishing a solid foundation for subsequent research and technological development. Furthermore, this innovative technology demonstrates considerable potential for diverse applications in both scientific research and clinical practice.

The rationale for choosing the ear as an ideal EEG monitoring site stems from its unique anatomical, structural, and physiological advantages. First and foremost, the ear possesses highly favorable anatomical properties. As a paired bilateral structure located on both sides of the skull, the ear region naturally ensures stable and consistent electrode–skin contact, as well as favorable acoustic impedance matching for reliable signal transduction [13]. In addition, the external auditory canal—measuring approximately 26 mm in length and 7 mm in diameter—allows safe and convenient device insertion, with a recommended depth limited to no more than 10 mm to avoid injury to the bony wall and tympanic membrane [1], supporting both usability and safety. Furthermore, from an electrophysiological perspective, cortical signals propagate to the ear canal after passing through cerebrospinal fluid, skull, and soft tissue layers, following an attenuation mechanism similar to that of conventional scalp EEG [13]. These structural and physiological merits establish the ear as a practical, minimally invasive, and stable location for long-term, wearable EEG monitoring.

Even so, this theoretical stability cannot be fully maintained in real long-term monitoring scenarios. Unavoidable perspiration and temperature changes cause gradual material deformation of in-ear devices, and prolonged compression leads to ear canal skin swelling [8,13]. Combined with substantial inter-subject anatomical variations, these problems trigger continuous contact impedance drift and signal instability. Such challenges related to electrode-skin contact have not been thoroughly resolved, and remain a major limitation for the large-scale deployment of long-term in-ear EEG systems [8,13].

Crucially, while in-ear EEG leverages the natural anatomical proximity of the ear to superficial temporal lobe structures, its inherent spatial sampling limitation strictly restricts recording coverage to limited lateral and inferior brain regions, thus rendering it poorly effective for investigating medial or frontal cortical activity. Meanwhile, the signal amplitude of in-ear EEG is inherently 2–5 times weaker than that of scalp EEG, primarily due to thicker intervening tissue and fewer effective neural sources oriented toward auricular sites, which collectively poses a core and fundamental SNR challenge.

## 3. Hardware Components, Design, and Performance of the In-Ear EEG System

### 3.1. Core Hardware Component of In-Ear EEG Systems: Electrodes

In-ear EEG electrode design constitutes a highly interdisciplinary endeavor that spans materials science, biomedical engineering, and human factors. Notably, in-ear EEG systems actively leverage state-of-the-art material innovations to simultaneously optimize long-term wearing comfort and effectively improve the quality of recorded neural signals [10]. Based on their structural and contact mechanisms, electrodes for in-ear EEG applications can be systematically classified into two primary categories: wet electrodes and dry electrodes [14,15].

#### 3.1.1. Wet Electrodes

Wet electrodes use electrolytic solutions, gels or pastes as ionic conductors to reduce the contact impedance between skin and electrodes, so as to improve signal quality. Furthermore, various implementations, such as paste-based, gel-based, sponge-based, and semi-dry designs, offer multiple alternatives for enhancing electrical contact between the skin and the electrode [16,17]. Specifically, silver chloride (AgCl) is a readily soluble salt that rapidly saturates the skin and forms a stable electrode–skin interface [18]. In fact, the first electrodes used in the development of the first in-ear EEG prototype were made of Ag/AgCl. Moreover, this type of electrode has superior properties, such as high biocompatibility, electrochemical stability, and optimal electrical conductivity, which are essential characteristics for obtaining high-quality EEG signals [4]. Typically, in terms of utilization, the Ag/AgCl electrode is often paired, to reduce the electrode–skin impedance for a high-quality EEG signal, with a conductive gel.

Overall, wet EEG electrodes present multiple advantages when compared with dry electrodes. Specifically, by forming a stable electrode-skin interface, the conductive gel, paste, or electrolyte serves to reduce contact impedance and improve the accuracy of recorded brain activity. Wet electrodes also have evident limitations: they require complicated installation and cleaning [14]. The conductive gel will evaporate over time, causing rising impedance, signal degradation and impedance drift during long-term monitoring. Furthermore, the conductive gel can also pose biocompatibility issues, including skin irritation and allergic reactions in some patients [19]. Benefiting from the bonding effect of conductive media, wet electrodes show low motion artifact susceptibility under slight head and jaw movements commonly occurring during ear EEG tests. Moreover, the need for regular maintenance and cleaning to prevent gel contamination or degradation represents a practical drawback, as it incurs additional effort and resource expenditure [20]. From the perspective of practical clinical usability, wet electrodes have established standardized operating protocols and reliable signal performance, making them the mainstream option for formal clinical diagnosis; nevertheless, their cumbersome operation and poor sustainability greatly limit their application in long-term home-based and portable clinical monitoring [20].

#### 3.1.2. Dry Electrodes

Dry electrodes are a major development trend of in-ear EEG systems. They require no conductive gel, which simplifies operation, improves wearing comfort, and reduces noise and artifacts in long-term brain monitoring [21]. Therefore, it is evident that dry electrodes for in-ear EEG can substantially improve the comfort and usability of in-ear EEG devices in daily scenarios. To realize high-performance dry in-ear EEG electrodes, a wide range of advanced materials has been extensively explored [22,23,24], including sintered silver/silver chloride (Ag/AgCl), silver-plated knit conductive fabric, conductive silicone rubber [23], gold wires, Ti-coated IrO_2_, polycarbonate coated with Ag, IrO_2_ conductive fabric, and carbon nanotube porins/polydimethylsiloxane (CNTP/PDMS) composites [5].Among these material candidates, conductive fabric electrodes are particularly widely adopted in viscoelastic earphone-style devices, owing to their excellent flexibility and reliable adaptability to mechanical deformation [25]. Furthermore, flexible conductive polymers have also been successfully applied in this field. For instance, Lee et al. [5] fabricated in-ear EEG electrodes using a CNTP (Ctube100, CNT Co., Republic of Korea)/PDMS (Sylgard 184, Dow Corning, Midland, MI, USA) composite, which exhibited both favorable electrical and mechanical properties.

However, dry electrodes have higher inherent impedance than wet electrodes, which reduces the SNR of collected EEG signals [6,26,27]. Their contact impedance is susceptible to sweat, contact pressure and individual physical differences, showing obvious dynamic changes. In addition, the signal fidelity of dry electrodes is more vulnerable to disturbances including contact pressure, body motion, perspiration, and individual anatomical differences in the ear canal [28]. Accordingly, dry electrodes show higher motion artifact susceptibility than wet electrodes, and body movement will further amplify signal distortion caused by unstable contact. Despite these drawbacks, dry electrodes remain the primary choice for in-ear EEG applications owing to their superior wearability, comfort, and portability [29,30,31]. With no volatile conductive medium inside, dry electrodes deliver outstanding long-term stability; their material properties and contact status remain stable during continuous multi-hour or even multi-day monitoring. Specifically, the absence of a conductive electrolyte layer at the electrode–skin interface represents a major limitation of dry electrodes relative to wet electrodes, resulting in elevated interfacial impedance and degraded signal quality [6,9,26,27,28]. Such issues typically introduce additional noise, lower the SNR, and diminish the precision in detecting weak brain signals. Furthermore, the relatively unstable contact between dry electrodes and the scalp or ear tissue renders the recorded signals more prone to motion artifacts, further impairing overall signal reliability.

The gel-free feature of dry electrodes not only simplifies the electrode setup process and enhances user operability but also enables superior reusability, as dry electrodes avoid the contamination and wear associated with gel-based interfaces. Such user-friendly characteristics significantly improve the technological accessibility of in-ear EEG systems [26]. In terms of practical clinical usability, dry electrodes feature simple operation and reusability, which are well suited for long-term ambulatory clinical monitoring, home follow-up and sleep assessment; however, the insufficient signal stability restricts their large-scale application in high-precision clinical diagnosis [14,26]. This user-friendly profile thereby increases technological accessibility and supports the expansion of in-ear EEG into long-term and at-home monitoring scenarios.

From a critical translational perspective focusing on long-term stability, impedance fluctuation, motion artifact resistance and clinical practicality, the two electrode modalities exhibit inherent trade-offs restricting their respective clinical deployment [14,17]. Wet electrodes degrade over hours due to gel evaporation and skin absorption, with sharp impedance increases; they resist motion artifacts well and suit short-term high-precision clinical diagnosis, but are unfit for long-term home monitoring and may cause skin irritation. Dry electrodes avoid gel-related failure, with mild impedance drift improvable via material design, yet gradual contact loosening from sweat and tissue changes impairs long-term stability [17,20]. Table 1 provides a concise summary of the key characteristics distinguishing dry and wet electrodes in in-ear EEG configurations [32].

To identify optimal electrode materials balancing conductivity, biocompatibility, flexibility and manufacturability for practical in-ear EEG, three mainstream material systems are summarized. First, IrO_2_-coated titanium features low impedance, good biocompatibility and long-term tissue stability [24]. Easy to machine, it is widely used in 3D-printed personalized in-ear electrodes for clinical SSVEP and epilepsy detection, though its high cost limits mass production. Second, silver-plated conductive fabric suits generic consumer devices well: it is flexible enough to fit diverse ear anatomies, provides stable conductivity and good biocompatibility, and supports low-cost processing [23,25]. It is the dominant material for universal earphone-style EEG electrodes, with only slight impedance rise under heavy sweating. Third, CNTP/PDMS composites combine the flexibility of PDMS and the stable conductive network of carbon nanotubes, and can be integrally molded for custom earpieces, while its sweat resistance still needs optimization for large-scale commercialization [5]. By comparison, conventional bulk Ag/AgCl only works for gel-assisted wet electrodes and is unsuitable for gel-free long-term wear. Rigid metals and common conductive silicones also fail to meet the flexibility demands for ear fitting.

### 3.2. Electrode Integration and Earpiece Design

#### 3.2.1. Customized (Personalized) Earpieces

Human ears vary greatly in anatomy, which brings challenges to the standardization of in-ear EEG electrodes and causes inconsistent signal collection among different users. Consequently, electrode designs must either possess sufficient flexibility to adapt to varied ear canal geometries or be fabricated in customized, subject-specific shapes. Researchers have increasingly employed three-dimensional (3D) printing technology to optimize EEG signal acquisition in the ear-EEG domain [33,34,35,36]. By leveraging the capabilities of additive manufacturing, complex shapes can be created, enabling a precise fit within the ear canal for improved electrode contact and signal quality [37,38]. For instance, Tabar et al. developed customized in-ear EEG electrodes by utilizing 3D printing, which improved both user comfort and signal accuracy [39]. Similarly, Yu et al. introduced a 3D-printed in-ear EEG device that integrates both the electrode and circuit board into a single printed structure [40]. This design has the potential to accommodate various ear shapes, enhancing usability across different individuals.

Despite these innovations, manufacturing conductive materials often requires additional steps like casting and chemical treatments, increasing production complexity. To address this issue, an in-ear EEG electrode with an extendable spiral structure was developed, using electrothermal actuation to conform to an individual’s unique ear canal geometry [8]. An alternative approach to creating personalized in-ear EEG electrodes involves integrating standard electrodes into custom-moulded soft earpieces. One implementation used a dry in-ear EEG system with IrO2-coated titanium electrodes embedded in elastic earmold silicone [36]. Another high-density configuration employed 15 electrodes per ear to achieve signal quality comparable to, or exceeding, scalp EEG in certain evaluations, such as auditory steady-state response (ASSR) and steady-state visual evoked potential (SSVEP), also using IrO2 as the electrode material [41].

To simplify earpiece fabrication, some researchers eliminated 3D modeling and instead used moldable, personalized materials matching the subject’s ear shape as the base for their in-ear EEG device. They also changed the electrode material to ease fabrication and improve compatibility with alternative earpiece materials. For instance, studies [42] employed moldable plastic beads (InstaMorph) for the fabrication of personalized earpieces.

#### 3.2.2. Generic Earpiece

As an innovative, robust, discreet, and noninvasive solution for monitoring brain activity outside the laboratory [43], in-ear EEG—which acquires signals directly from the ear canal—has attracted growing interest. However, anatomical variability across individuals poses a major challenge for standardized in-ear EEG electrodes, frequently leading to unstable signal quality. Consequently, electrodes must be either sufficiently compliant to adapt to unique ear canal contours or fabricated in subject-specific geometries. To address these issues, generalizable in-ear EEG electrodes have been developed, designed to accommodate a wide range of individual ear anatomies [18,44]. Such electrodes are typically fabricated from elastic materials, allowing them to deform and adapt to individual ear canal geometries [44]. This adaptability ensures a stable interface, thereby improving both signal quality and user comfort during extended wear [30]. This approach benefits from simple manufacturing, offering a cost-effective, one-size-fits-all solution.

In 2013, the pioneering team in in-ear EEG technology introduced one of the first generic designs for such an in-ear EEG device [45]. Their design was based on an earplug with a conical geometry, constructed from biocompatible silicone rubber. In recent years, a number of innovative and effective designs for generic in-ear EEG devices have been proposed, demonstrating both innovation and efficacy [26,46,47,48]. For instance, A foam substrate is used in one design to confer adaptability to the ear canal’s shape. This enhances the fit and stabilizes the signal [49]. This design incorporates a two-electrode, two-microphone configuration for stimulus application. The selected material effectively mitigates motion artifacts caused by both minor and substantial mechanical deformations of the ear canal walls [49]. Kaveh et al. [46] employed a 3D scan of the in-ear anatomy to create a generic earpiece design. The design features four outward-extending cantilevers, each functioning as an individual electrode to apply pressure to the ear canal, complemented by two larger electrodes positioned on the concha for reference and ground connections. In their finalized version [47], the earpiece body was 3D-printed using a flexible resin (FLTOTL4, Formlabs, Somerville, MA, USA) to improve comfort. In 2022, Paul et al. [48] presented the weDAQ system, a wireless platform for acquiring multiple electrophysiological signals, including in-ear EEG. The system incorporates a generic in-ear EEG design consisting of two planar ‘crocodile’ PCBs that assemble into a 3D structure with surface-mounted electrodes. To overcome the protracted manufacturing time and limited lifespan of personalized in-ear EEG electrodes, Liang et al. [50] introduced a method that leveraging impressions to fabricate a generalized device based on averaged ear measurements across three key dimensions: the aperture, isthmus, and length.

In summary, personalized and generic earpieces differ greatly in four key aspects. Personalized earpieces fit individual ear anatomy precisely, with stable electrode contact, high signal repeatability and good long-term comfort. But customized design and manufacturing lead to high costs and low production scalability [40,49]. In comparison, universal earpieces are suitable for mass production with low costs, yet their fixed structure cannot adapt to different ear shapes, resulting in unstable contact, poor repeatability and discomfort after long wear [26,40].

Inspired by the structural design of commercially available wireless earbuds, most current generic and customized in-ear EEG devices adopt an intra-aural wearing form. However, compared with circumaural head-mounted devices, in-ear solutions are inherently constrained by the narrow anatomical space of the ear canal. Long-term wearing will easily cause ear canal compression, local soreness and foreign body sensation, which significantly reduces user comfort and compliance during all-day or multi-day continuous monitoring. This ergonomic defect has become one of the key factors restricting the large-scale popularization and clinical application of in-ear EEG. Optimizing the overall structure, material flexibility and contact pressure of earpieces has therefore become an important direction to balance signal stability and long-term wearing experience for subsequent product iteration.

Inter-subject ear anatomical differences severely degrade cross-participant signal repeatability, causing inconsistent electrode contact and fluctuating impedance across distinct users. Two mainstream practical solutions are available at present: one relies on 3D scanning and personalized 3D printed earplugs to achieve individual matching and stable contact; the other adopts elastic generic earpieces with deformable cantilever or foam structures to adapt diverse ear anatomies for improved inter-subject signal consistency.

### 3.3. Electrode Configuration and System Architecture of In-Ear EEG

Most in-ear EEG studies utilize a limited number of channels, typically fewer than eight per earpiece, a design choice that primarily stems from the constrained space within the ear canal. Consequently, the electrodes must also be small to fit snugly within the ear, thereby making the fabrication process more intricate [51]. The complexity of this situation becomes particularly noticeable when dealing with handcrafted electrodes. In addition to the design and material of the device, the number of EEG channels is an important factor to consider when designing an in-ear EEG acquisition system [29,52]. Despite limited spatial coverage, the in-the-ear potential field can still be measured at multiple points to provide spatial information. The pioneering work on a high-density in-ear EEG device was reported by Kappel et al. in 2017 [41]. Each personalized earpiece featured 15 uniformly embedded electrodes on each side, constructed from circular titanium pins coated with IrO2. Another variation of a high-density in-ear EEG device was introduced by Paul et al. in 2019 [53,54]. This device featured 17 small Ag/AgCl electrodes fitted into each personalized earpiece, rather than the IrO2 electrodes used in earlier studies.

**Figure 3:**
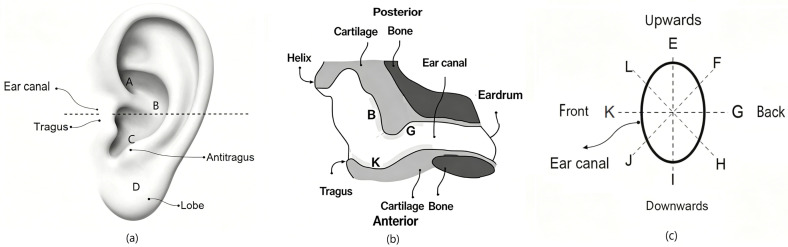
Ear electrode naming convention for the left ear. (**a**): External ear sketch marking regions A–D. (**b**): Axial cross-section showing positions B, G, and K. (**c**): Sagittal ear-canal cross-section with electrode labels. Naming follows vertical direction, not insertion depth. Adapted from [3].

Though no universally accepted international standard exists for in-ear EEG electrode nomenclature and arrangement, the original in-ear EEG research group’s [3] methodology is notable for its ingenuity and meticulous electrode positioning (Figure 3).This specific approach designates each electrode with the identifier EXY, where X represents either L or R, indicating the left or right side of the ear, respectively, and Y denotes the electrode’s position using letters from A to L (e.g., ERA, ERB,…, ERL). Among these designations, A, B, and C mark the upper, middle, and lower concha regions, respectively; D corresponds to the earlobe.

The reference and ground electrodes (EXA, EXB, EXC) are typically positioned on the concha, a location that is farther from the brain than the ear canal. Nonetheless, numerous studies employ additional electrodes with separate cables attached to the earlobe, around-ear area, or mastoid, which function as reference and ground points [55]. This practice leads to their system not aligning with a genuine in-ear EEG setup.

Practically, hybrid layouts combining intra-canal sensing electrodes and externally mounted reference/partial electrodes (on concha, mastoid or retroauricular area) have been widely validated to boost overall signal performance against fully in-ear configurations [4,6]. External reference electrodes stabilize baseline potential and suppress common-mode noise, whereas extra outer electrodes expand sampling coverage beyond limited temporal cortical regions, lowering overall signal distortion and electrode-skin impedance [8,17]. However, this structural improvement comes at the cost of compromised wearing invisibility and compactness, departing from the core design advantages of fully embedded in-ear EEG for concealed daily wearable monitoring, which explains why all-intra-canal electrode design remains a mainstream research direction for consumer-oriented ear-EEG devices despite its inferior raw signal quality [13,15].

### 3.4. Performance Comparison of In-Ear EEG Hardware with Conventional Scalp EEG Hardware

Conventional scalp EEG uses multiple electrodes to cover the entire cerebral cortex and achieve high spatial resolution and high-fidelity signal collection. For routine long-term monitoring tasks such as sleep and drowsiness detection that do not require full cortical mapping, in-ear EEG can meet the demand. It is a practical alternative that balances performance and usability [9,56]. In contrast, in-ear EEG systems generally employ a sparse electrode array consisting of only 2–6 channels, which are typically placed within the external auditory canal [57,58]. Owing to this spatial arrangement, neural activity recordings are inherently restricted to brain regions proximal to the ear [57,58]. As a direct result, this arrangement inherently yields poorer spatial resolution relative to conventional scalp EEG. Even so, in-ear EEG’s advantages in portability, long-term wearability, and ease of use make it a valuable alternative for scenarios where scalp EEG is not feasible, such as home-based monitoring or mobile neurophysiological assessment [59,60].

Regarding motion artifacts, conventional scalp EEG demonstrates greater robustness to motion during ambulatory activities. By contrast, in-ear EEG signals are highly susceptible to artifacts induced by jaw and head movements—artifacts that can substantially compromise signal integrity [9,58]. In ambulatory scenarios such as speaking, chewing and walking, in-ear EEG is highly vulnerable to jaw muscle artifacts, which cause obvious signal fluctuations. Strenuous exercise further loosens electrodes and triggers a sharp rise in contact impedance, whereas mild walking only results in minor signal drift [2,8]. Compared with conventional scalp EEG, in-ear EEG is less affected by ocular artifacts thanks to its enclosed in-ear structure. Although flexible earpiece designs and specialized artifact suppression algorithms can enhance signal stability to some extent, the overall performance still degrades significantly under intense physical activity. Nevertheless, recent advances in signal processing algorithms have helped mitigate these artifacts, further enhancing in-ear EEG’s competitiveness as a substitute for scalp EEG in real-world, dynamic environments.

Beyond artifact susceptibility, the two methods also exhibit considerable differences in key signal quality parameters: electrode-skin impedance, signal amplitude, and signal-to-noise ratio (SNR) all vary markedly between conventional scalp EEG and in-ear EEG, further underscoring their divergent performance profiles [57,61]. For a systematic overview of these distinctions, a thematic comparison of conventional and in-ear EEG is provided in Table 2. Overall, while scalp EEG remains the gold standard for high-spatial-resolution applications, in-ear EEG’s unique advantages in comfort, portability, and long-term monitoring make it a promising alternative that can complement or even replace scalp EEG in a wide range of practical and clinical scenarios [61].

Conventional scalp EEG features outstanding precision and anti-interference capability for rigorous clinical diagnosis and high-accuracy BCI tasks, while in-ear EEG excels in wear comfort and long-duration operational stability suited to ambulatory daily health monitoring, at the cost of compromised spatial resolution and signal precision [9]. From a developmental perspective, this study anticipates that iterative improvements in sensing hardware and intelligent signal processing methodologies will gradually mitigate the inherent performance discrepancy between the two recording modalities, rendering in-ear EEG an essential complementary option to conventional scalp EEG across multifarious practical application domains [13,51]. Despite the compact and wearable form of in-ear EEG systems, critical limitations remain in electrode and system design. Overall, in-ear EEG still faces an unresolved trade-off between wearability, electrode–skin contact quality, and manufacturing scalability, which severely restricts its long-term stability and clinical translation.

Collectively, constrained by reduced signal amplitude and limited spatial sampling range focused on temporal brain regions, in-ear EEG cannot completely replace conventional scalp EEG for comprehensive clinical diagnosis requiring full-cortex mapping [9]. Nevertheless, abundant experimental and clinical data from recent investigations provide credible evidence to support its partial substitution value in specific clinical scenarios [18,42]. For temporal lobe epilepsy long-term follow-up, overnight home sleep assessment, driver fatigue real-time warning and daily mental state continuous monitoring, in-ear EEG achieves detection accuracy consistent with scalp EEG and can serve as an economical, wearable alternative for out-of-hospital screening and prolonged ambulatory surveillance, while formal definitive clinical diagnosis still depends on standard scalp EEG recordings [39,44].

Compared with other mainstream wearable EEG modalities, in-ear EEG owns unique pros and cons: Around-ear (periauricular) EEG gains broader temporal coverage yet is prone to pull-induced artifacts from ear-edge skin shift; forehead EEG easily captures frontal brain signals but suffers severe eye-blink and facial muscle interference [9,14,55]. Ultra-thin tattoo and epidermal electrodes achieve ultra-low skin impedance and outstanding anti-motion performance for long-term monitoring, whereas they require repeated skin adhesion and are inconvenient for daily wearing replacement [51]. Benefiting from ear canal enclosed fitting, in-ear EEG avoids hair occlusion and ocular artifacts, featuring superior concealment for all-day wearable monitoring, but its signal is heavily disturbed by jaw/chewing artifacts and limited to temporal lobe sampling compared with the above alternatives [9].

## 4. Signal Processing and Artifact Removal

In long-term monitoring, in-ear EEG can resist motion and environmental noise to a certain extent [44,62], but artifacts still restrict its practical application. Muscle activities such as swallowing, speaking and head movement will seriously interfere with in-ear EEG signals. In addition, sensor micro-movements and sweat will change the contact state between electrodes and skin over time, leading to obvious signal fluctuations [51,63]. Environmental electrical noise, instrument interference and electrode displacement will also degrade signal quality. Collectively, these represent key issues that must be resolved to improve the practicality of in-ear EEG systems [10,64].

Beyond artifacts, in-ear EEG signals are inherently weak and spatially restricted: they predominantly capture temporal lobe activity with amplitudes approximately 10% of those from scalp EEG, while signals from the occipital and parietal lobes are barely detectable. Furthermore, the limited number of recording channels and restricted scalp coverage further constrain its spatial resolution [51]. Another critical limitation is that most existing denoising algorithms are directly migrated from scalp EEG without tailored design for ear-EEG’s unique artifact patterns, resulting in insufficient noise suppression [63]. Therefore, advanced signal processing techniques are essential to improve the quality of ear-EEG recordings [51].

### 4.1. Hardware Strategies for Artifact Reduction in Ear EEG

Hardware design serves as the most fundamental and effective strategy for suppressing artifacts in-ear EEG systems, where comprehensive optimizations are systematically focused on four core modules: earpiece structure, electrode–skin interface, analog readout circuits, and wireless transmission. Initially, custom-fitted earpieces were widely employed to firmly fix electrodes inside the ear canal, which can effectively reduce motion-induced interference during body movements. To meet the requirements of practical commercial applications, generic reusable earpieces constructed from memory foam and flexible polymers have been further developed, which ingeniously balance conformal fitting stability, long-term wearing comfort, and mechanical suppression of motion artifacts [65,66]. In terms of electrode and circuit improvements, dry active electrodes integrated with impedance bootstrapping, direct current (DC) servo loops, and active shielding can effectively mitigate baseline drift, contact instability noise, and wire-related interference that commonly corrupt ear-EEG signals [21,67]. Furthermore, wireless transmission schemes represented by Bluetooth Low Energy (BLE) are adopted to completely eliminate motion artifacts caused by long connecting cables, thereby significantly enhancing signal stability and reliability for daily wearable scenarios [46].

### 4.2. Advanced Signal Processing Techniques for Artifact Removal in Ear EEG

Advanced signal processing plays an indispensable and critical role in eliminating residual artifacts in ear-EEG systems, especially under the practical constraints of limited channels, low amplitude, and low SNR. First of all, conventional denoising methods from general EEG studies have been effectively adapted and optimized for in-ear EEG applications. Adaptive filtering incorporated with motion references such as accelerometers can selectively cancel motion-induced noise components in continuous recordings [51]. In addition, blind source separation (BSS) methods, mainly represented by independent component analysis (ICA) and canonical correlation analysis (CCA), can robustly separate neural signals from motion and muscle artifacts by identifying statistically independent or correlated signal sources [64,68,69]. Moreover, signal decomposition algorithms, including wavelet transform and empirical mode decomposition (EMD), provide reliable solutions for single-channel in-ear EEG artifact suppression through decomposing mixed signals into time–frequency components or intrinsic mode functions [64,70]. To further overcome the limitation of spectral overlap, hybrid frameworks such as EMD–ICA and Wavelet–ICA significantly enhance denoising performance when artifacts and neural signals are highly overlapped in the frequency domain [68]. A small set of hybrid EMD–ICA and wavelet-based algorithms have been specially optimized for in-ear-specific jaw muscle artifacts, while most existing artifact removal solutions remain adapted from scalp EEG processing pipelines.

For the specific jaw and muscle artifacts inherent to in-ear EEG, blind source separation and optimized decomposition algorithms exhibit the most promising suppression performance. Among them, ICA and CCA can effectively separate muscle-related components from original neural signals; hybrid EMD–ICA and Wavelet–ICA further resolve frequency overlap between brain signals and jaw artifacts, outperforming single algorithms [68,69]. Besides, motion-aided adaptive filtering with acceleration reference also delivers satisfactory real-time suppression for intermittent jaw movement noise and has been widely validated in existing in-ear monitoring research [70].

### 4.3. AI-Based Signal Processing Strategies for Artifact Suppression and Signal Quality Improvement in Ear EEG

In recent years, artificial intelligence (AI) has become a core solution for artifact removal and signal optimization of in-ear EEG. Various AI technologies enable automatic end-to-end processing without manual intervention, which is highly suitable for long-term wearable monitoring scenarios. According to algorithm categories, existing AI-based processing methods are sorted and summarized as follows.

#### 4.3.1. Conventional Machine Learning and Federated Learning

Classical machine learning algorithms (e.g., Random Forest and Logistic Regression) are widely used for adaptive artifact removal. They can also realize automatic sleep staging, motor imagery recognition, speech imagery recognition and emotion classification based on in-ear EEG signals. As an effective distributed learning paradigm, federated learning can be combined with the above classical models to reduce inter-subject variations, strengthen the generalization capability of classification models across different users, and facilitate the practical deployment of monitoring systems [71,72].

#### 4.3.2. CNN-Based Approaches

Convolutional Neural Networks (CNNs) serve as mainstream deep learning architectures for in-ear EEG processing. They can automatically extract discriminative features from high-dimensional raw EEG data [73,74,75]. Compared with traditional signal transformation methods such as short-time Fourier transform (STFT) and discrete wavelet transform (DWT), CNNs achieve superior denoising performance and can identify and suppress multiple types of artifacts without manual feature engineering [76,77]. Combined with generative models, CNN-based frameworks can recover high-fidelity EEG signals from artifact-contaminated raw data, simplifying preprocessing procedures [73,74].

#### 4.3.3. LSTM-Based Approaches

Long Short-Term Memory (LSTM) networks excel at capturing complex temporal dependencies of sequential EEG data. This characteristic makes them effective for detecting context-aware artifacts caused by body movement and eye movement in dynamic daily scenarios. LSTM and its variants are also extensively adopted in lightweight edge computing tasks such as sleep staging for wearable in-ear EEG devices [11,78,79].

#### 4.3.4. Autoencoder-Based Approaches

Dual Path Autoencoders (DPAE), U-Net and other autoencoder-derived architectures are dedicated to separating effective brain signals from complex artifacts. These models further improve the accuracy of artifact suppression and overall signal reliability, and have become important supplements to existing denoising frameworks for in-ear EEG [80].

At present, AI classification models for in-ear EEG suffer from poor reproducibility and cross-dataset generalization. Most existing models are trained and validated using small, single-center datasets collected from healthy participants under laboratory conditions. Large inter-individual differences in ear anatomy lead to unstable electrode contact and varying signal characteristics across users, while inconsistent chewing and facial movement artifacts further exacerbate performance discrepancies. When pre-trained models are applied to unseen user groups or independent datasets, their classification accuracy drops significantly. Additionally, the lack of standardized public in-ear EEG databases makes it difficult to conduct unified benchmark tests and replicate experimental results. Federated learning presents a promising solution to address these generalization limitations; however, its practical application still requires large volumes of real-world data from diverse populations to achieve reliable performance improvements.

Most existing signal processing and artifact removal methods for in-ear EEG are directly adopted from scalp EEG without customization, resulting in limited performance on jaw EMG and motion artifacts. Many advanced artifact suppression techniques rely on extra sensors, increasing system complexity and compromising wearability. Collectively, the lack of standardized, lightweight, in-ear-specific pipelines impairs signal stability, reproducibility, and real-world utility for long-term ambulatory monitoring.

## 5. Applications of In-Ear EEG

### 5.1. Brain-Computer Interfaces (BCIs) Based on In-Ear EEG

BCI technology builds a direct connection between the brain and machines to replace, repair, assist or enhance brain functions. A standard BCI system consists of four modules: signal acquisition, preprocessing, feature extraction & classification, and output equipment [81]. For medical applications, BCIs demand long-term continuous monitoring—such as for epilepsy and migraine—and real-time control of assistive devices including prosthetics, robotic arms and wheelchairs in daily scenarios. Wearability is thus essential for the long-term deployment of BCI systems. Compared with invasive approaches, in-ear EEG based BCI permit non-invasive signal recording outside laboratory environments, rendering them highly suitable for wearable daily use [58].

In 2011, Looney et al. [1] developed an ITE electrode system for wearable ear-EEG recording. These ITE electrodes exhibited strong correlation and coherence with conventional scalp electrodes, successfully capturing key EEG features including the auditory steady-state response (ASSR), alpha attenuation response (AAR), and P300 potentials. Consequently, these early findings clearly highlighted the significant potential of in-ear EEG for practical BCIs applications [13]. Subsequently, a growing number of comparative studies has further validated that in-ear EEG can reliably capture effective neural activity associated with both exogenous stimulation and endogenous imagination, ranging from steady-state evoked potentials (SSVEPs) and event-related potentials (ERPs) to motor imagery, speech imagery, and visual imagery [6]. Therefore, in-ear EEG has broad application prospects in auxiliary control, silent human-computer interaction, neural rehabilitation and intelligent hardware [81].

#### 5.1.1. Steady-State Responses (SSRs): ASSRs and SSVEPs

SSRs are periodic neural oscillations induced by external periodic sensory stimulation [82,83]. Featuring stable frequency-locking behavior and a high SNR, SSRs stand as one of the most widely used control signals in BCI systems.

In terms of ASSRs, in-ear EEG holds inherent natural advantages in signal acquisition, owing to its close anatomical proximity to the auditory cortex and auditory pathways [84,85]. Similarly, SSVEPs are primarily generated in the occipital lobe. Despite the spatial distance between the periauricular region and the occipital area, in-ear EEG can still effectively capture valid SSVEPs signals through rational electrode placement and dedicated signal processing algorithms, enabling reliable visual-stimulation-based BCI control [86,87].

Numerous studies have consistently verified that in-ear EEG can stably induce and extract ASSR signals, particularly the 40 Hz ASSR—a signal commonly utilized in clinical hearing evaluation [88,89]. Pioneeringly, Kidmose et al. [65] were the first to confirm that the ASSR signal collected by in-ear electrodes possesses a SNR equivalent to that of temporal scalp electrodes; while its amplitude is only slightly reduced, this reduction does not compromise classification judgment or signal utility. Subsequently, a series of follow-up studies employing dry electrodes, flexible electrodes, and viscoelastic earplugs have yielded consistent conclusions: both in-ear and periauricular structures can effectively capture 40 Hz and 80 Hz ASSR signals, and their SNR is sufficiently high to support reliable automatic detection [65]. Furthermore, in terms of electrode configuration, the ear Fpz, intra-ear, and cross-ear reference methods all enable the acquisition of statistically significant ASSR signals. Notably, among these configurations, the dry-contact flexible electrode achieves a higher average SNR than ordinary universal in-ear devices [26], highlighting the impact of electrode design on ASSR signal quality.

In terms of SSVEP signal acquisition using in-ear EEG, early studies found that the SSVEP amplitude collected from the ear area is lower than that from the occipital scalp. However, the introduction of signal enhancement algorithms—such as error correction regression (ECR) and phase optimization—can significantly improve recognition accuracy. Specifically, Ahn, J.W. et al. [90] developed a single-channel in-ear SSVEP BCI system using conductive rubber electrodes (Model 8500370; Sanibel Supply, Hillerød, Denmark) achieving an accuracy of 79.9% ± 13.1% and an information transmission rate of 11.03 ± 4.18 bits/min. Subsequently, Liang et al. [91] further optimized the phase difference of left-right visual field stimulation, enabling the superposition enhancement of in-ear EEG SSVEP signals and a significant improvement in classification accuracy. Currently, online four-class SSVEP BCIs based on in-ear EEG can stably achieve an average accuracy of over 85%, which is close to the performance of traditional scalp EEG and applicable to portable, concealed, and hands-free control scenarios [86]. Additionally, Sun, Y. et al. [92] proposed a Multi-layer Ear-Scalp Distillation (MESD) framework that transfers knowledge from scalp EEG to ear EEG, effectively enhancing the classification performance of ear-EEG-based SSVEP and increasing its practical application value—achieving 81.1% SSVEP classification accuracy within 1 s and further boosting the practicality of in-ear EEG for BCI applications.

#### 5.1.2. Event-Related Potentials (ERPs) in In-Ear EEG-Based BCIs

ERPs refer to transient neural potential changes elicited by specific events or stimuli, reflecting high-level cognitive processes including attention, judgment, and memory, and thus represent important signal sources for BCIs. Among these components, the P300 potential and auditory attention decoding-related ERPs have been the most extensively investigated in-ear EEG research [93].

The P300 potential is a positive peak occurring approximately 300 ms after target stimulation, and it has been widely applied in BCIs such as spelling systems and command selection. Notably, in-ear EEG is capable of capturing distinct P300 signals under both visual and auditory oddball paradigms. Specifically, Debener et al. [94] first employed cEEGrid periauricular electrodes to record EEG during auditory oddball tasks, achieving a P300 classification accuracy of 70.92%. Subsequently, Bleichner et al. [95] developed a visual P300 speller using a hybrid electrode configuration combining scalp (Dreve Otoplastik GmbH, Unna, Germany), periauricular, and in-ear electrodes, attaining an accuracy of 88% and an information transfer rate of 8.33 bits/min. Furthermore, Kaongoen et al. [96] established an auditory P300 BCI system based on ear-EEG, enabling covert command recognition without visual engagement and delivering a more natural user experience.

In contrast to conventional scalp EEG-based BCIs, in-ear EEG P300 systems do not require users to fixate on a screen, thereby supporting non-visual control and making them particularly well-suited for daily, covert applications.

#### 5.1.3. Active Imagery-Based In-Ear EEG BCIs

Endogenous imagery-based BCIs operate independently of external stimulation; instead, users generate control commands directly through voluntary mental imagination, granting them the highest degree of flexibility among BCI modalities. A key representative of this paradigm is active brain imagery, which describes the stimulus-free neural activity elicited by self-initiated cognitive tasks [97], encompassing major subcategories including motor imagery, speech imagery, and visual imagery.

Against this background, in-ear EEG has achieved remarkable progress in decoding these endogenous imagination patterns, enabling truly stimulus-independent BCI. As early evidence, Merrill et al. [98] first demonstrated reliable classification of multiple mental tasks using ear-EEG, achieving 85.4% average accuracy and over 90% accuracy in half of the participants. Building on this achievement, they subsequently developed a custom ear-EEG “passthought” authentication system that reached 99.82% accuracy, while personalized fitting further enhanced its anti-imposter robustness.

Motor imagery (MI) mainly induces mu and beta band event-related desynchronization (ERD) by imagining limb movements, and thus is widely used in neurorehabilitation and auxiliary control [99,100,101,102]. Specifically, Kim et al. [103] selected channels near the ear from the standard 10–20 system scalp EEG, and found that the four-class motor imagery accuracy was equivalent to that of the traditional layout, which suggests that in-ear EEG devices could be used for measuring motor imagery paradigms. In addition, Wu et al. [104] developed a custom in-ear EEG device with Ag/AgCl electrodes, and achieved effective two-class motor imagery recognition using the EEGNet model. Notably, although in-ear EEG motor imagery accuracy is slightly lower than that of the full-scalp layout, it can reach more than 70% after fine-tuning and algorithm optimization, which fully meets the needs of auxiliary control and rehabilitation training. Most importantly, the advantage of in-ear EEG is that it is comfortable to wear and does not affect daily activities, making it particularly suitable for long-term home rehabilitation training.

Speech imagery (SI) refers to the neural activity generated when the user imagines pronunciation without making a sound, which is the core of silent communication BCI. As the language center is located in the temporal lobe, in-ear EEG has natural advantages in collecting speech imagery signals. Kaongoen et al. [105] pioneered the investigation of in-ear EEG for speech-imagery BCIs, demonstrating its viability as a practical alternative to scalp-EEG. Their findings underscore the potential of this approach to accelerate the development of unobtrusive BCI systems for daily life. Subsequently, they developed a wearable ear-EEG system enabling users to control home appliances via silent speech imagery. All 11 participants successfully decoded commands offline, surpassing chance-level accuracy. In online tests, top performers achieved rapid control, with the best user reaching 85% accuracy at 3.79 s per command [106]. This technology breaks through the limitation that traditional BCIs cannot realize silent interaction, and can be applied to special scenarios such as silent communication, auxiliary speaking, and intelligent voice interaction without disturbing others.

Visual imagery (VI) and visuomotor tracking are also important branches of in-ear EEG-based endogenous BCIs [107], further expanding the scope of stimulus-independent brain control enabled by in-ear EEG technology. Specifically, Kuatsjah et al. [108] developed a two-channel wireless in-ear EEG system, which could effectively distinguish between the user’s resting state and visuomotor tracking state, with an accuracy significantly higher than the chance level—providing initial evidence for the feasibility of ear-EEG in visuomotor-related BCI applications. In a more in-depth study, Todoroki, S. et al. [109] integrated the EEG Conformer model with explainable AI (XAI) to decode imagined movement speeds, while simultaneously revealing the associated temporal–spatial patterns, characteristic frequency bands, and relevant cortical regions.

Taken together, these findings confirm that motor imagery speed is decodable using in-ear EEG (despite limited performance), and its neural encoding relies on specific oscillatory patterns, dedicated cortical areas, and SSRs.

Nevertheless, in-ear EEG signals are relatively weak and particularly vulnerable to jaw movements and muscle artifacts, which significantly compromises the robustness of BCI decoding. Furthermore, existing feature extraction and classification algorithms tailored for in-ear EEG are still inefficient and insufficiently mature for real-world tasks. Collectively, unstable signal quality, immature decoding algorithms, and incomplete brain region mapping jointly restrict the practical performance and large-scale deployment of in-ear EEG in BCI applications.

Overall, existing in-ear EEG-based BCI performances remain largely experimental rather than robust enough for routine daily deployment. Most reported classification accuracies are acquired under constrained laboratory environments with fixed postures and controlled surroundings [105,106]. In real daily scenarios including talking, chewing and walking, frequent jaw muscle artifacts and variable electrode contact lead to obvious accuracy degradation. Though partial speech-imagery and SSVEP-BCI achieve acceptable offline results, stable long-term practical control across diverse users is not yet realized, consistent with related summary in existing literature [81]. Further optimization of anti-artifact algorithms and personalized hardware is essential to boost real-world robustness.

### 5.2. Brain State Monitoring

A core advantage of in-ear EEG is its unique capability to enable long-term, ambulatory, and naturalistic brain state monitoring. Comfortable, portable, and easy to operate independently, in-ear EEG can continuously collect brain activity data in daily scenarios. This distinctive advantage has facilitated its wide application in several key fields, including sleep monitoring, epilepsy detection, drowsiness monitoring, and emotion recognition, which are elaborated as follows.

#### 5.2.1. Sleep Monitoring and Sleep Quality Improvement

The ear is anatomically close to the temporal lobe, so in-ear EEG can collect sleep-related neural signals such as slow waves and sleep spindles, which are key indicators for sleep stage division. Compared with traditional scalp EEG, in-ear EEG provides a viable alternative for sleep monitoring and staging [62]. Sleep stages typically include wakefulness (W), non-rapid eye movement (NREM) sleep (encompassing N1, N2, N3 stages) and rapid eye movement (REM) sleep, which are core indicators for assessing sleep quality and physiological state.

As an early pioneering investigation, Looney et al. [110] verified that in-ear EEG can acquire brain activity highly consistent with scalp EEG. Specifically, they evaluated in-ear EEG in three sleep classification tasks (N2/N3, N1/W, NREM/W), obtaining Kappa coefficients of 0.65 and 0.60, which provided early evidence supporting the signal consistency between in-ear EEG and scalp EEG. Subsequently, Palo et al. [111] extended this line of inquiry by conducting a head-to-head comparison between in-ear EEG and polysomnography (PSG) across ten subjects. In their study, 30-s EEG epochs were subjected to time and frequency domain feature extraction for hypnogram reconstruction, with similarity quantified via the Jensen–Shannon divergence-based feature similarity index (JSD-FSI). The results corroborated the high concordance of in-ear EEG with PSG signals across sleep stages (JSD-FSI: W 0.61 ± 0.06; NREM 0.60 ± 0.07; REM 0.51 ± 0.08), thereby validating in-ear EEG as a reliable alternative for objective sleep monitoring. De Fazio et al. [11] employed a multimodal EEG system (incorporating forehead and in-ear canal recordings) and proposed a two-stage attention-based LSTM model for sleep staging. The system achieved a sleep staging accuracy of up to 97.9%, validating the feasibility of multimodal EEG for high-precision sleep staging and providing a technical basis for low-cost, home-use wearable sleep monitoring solutions.

Regarding home sleep monitoring, Tabar et al. [44] validated a household in-ear EEG system via multi-subject and multi-night trials. Comparative evaluation against standard PSG yielded a Kappa coefficient of 0.71, demonstrating that in-ear EEG is a viable alternative to conventional PSG for at-home sleep staging assessment. Moreover, Borges et al. [112] extended comparisons to wrist actigraphy (ACT). All participants underwent simultaneous PSG and in-ear EEG recording, with additional ACT data acquired in healthy subjects. Outcomes revealed strong agreement between in-ear EEG and PSG for both sleep metrics and staging (Kappa > 0.80), with performance exceeding that of ACT. These findings further highlight the potential of in-ear EEG in streamlining home sleep evaluation.

PSG is the gold standard for clinical sleep detection. Current in-ear EEG systems are mature enough for household sleep screening and can complete reliable sleep stage scoring [39,112]. However, it cannot collect comprehensive physiological indicators for professional clinical diagnosis, so its performance in full sleep parameter detection is inferior to PSG.

Beyond sleep monitoring, in-ear EEG also exhibits strong feasibility and potential for sleep quality improvement via intervention strategies. For instance, Hestermann et al. [113] developed a wireless in-ear EEG device enabling automated closed-loop sleep modulation using combined 3 Hz binaural beats and ASMR to prolong deep sleep. The intervention group received staged stimulation, while the control group received only ASMR. The intervention group exhibited elevated N3 sleep duration and reduced N2 sleep duration.

#### 5.2.2. Epilepsy Monitoring

Epilepsy is a common neurological disorder characterized by recurrent seizures, often accompanied by involuntary movements and even loss of consciousness. In-ear EEG offers notable advantages including concealment, comfort, and low susceptibility to muscular artifacts, making it a promising tool for long-term ambulatory epilepsy monitoring [114].

Over the years, research has demonstrated its high sensitivity in seizure detection (especially for temporal and frontal lobe epilepsy), equivalent or superior performance to scalp EEG in capturing ictal and interictal epileptiform discharges, and good long-term wearing tolerance even in Alzheimer’s disease (AD) patients. For example, Musaeus et al. [36] conducted a longitudinal feasibility study involving ten AD patients to evaluate in-ear EEG for long-term monitoring. All patients wore the ear-EEG for at least 24 h, yielding acceptable-quality data after preprocessing, with only mild adverse events reported and partial patient dropout due to discomfort.

Alongside technological and algorithmic innovations, the clinical value of standalone in-ear EEG systems has been consistently demonstrated in clinical practice. Joyner et al. [115] carried out a clinical feasibility study in 20 patients with refractory focal epilepsy, verifying that in-ear EEG achieves high consistency with scalp EEG for seizure detection and performs better than conventional ambulatory monitoring. These findings validated the clinical value of in-ear EEG and confirm its applicability to long-term home-based epilepsy management. Beyond epilepsy-specific monitoring, Zeydabadinezhad et al. [116] assessed a custom-fit in-ear wearable for ambulatory EEG recording, demonstrating its reliable signal quality and superior wearing comfort. Their work further extends the application potential of in-ear EEG to a broader spectrum of neurophysiological monitoring scenarios.

Existing in-ear EEG-based epilepsy monitoring still has obvious restrictions on seizure subtype classification and false positive control. Limited to temporal-lobe-only signal acquisition, the device cannot adequately capture cortical discharges originating from frontal and parietal regions, leading to poor identification of non-temporal seizure subtypes. Besides, frequent jaw and facial muscle artifacts easily induce false-positive seizure alerts under daily activities, restricting stable long-term outpatient monitoring performance, as also reflected in existing clinical trial outcomes [114,115].

Recognizing the limitations of single-modal monitoring, researchers have increasingly turned to multimodal integration to enhance seizure detection accuracy. For instance, Vandecasteele et al. [117] combined ECG with limited-channel EEG to achieve improved detection accuracy. Similarly, Nielsen et al. [118] developed a wearable system integrating in-ear EEG, ECG, and accelerometry, enabling reliable out-of-hospital monitoring with high sensitivity. In parallel research, Bhagubai et al. [119] incorporated ECG into behind-the-ear EEG, while Zhang et al. [120] and Zibrandtsen et al. [121] fused ear-EEG with EMG and accelerometer signals. Overall, these diverse multimodal strategies all effectively enhanced seizure detection performance and collectively demonstrated the value of multimodal in-ear EEG in ambulatory neurological monitoring.

#### 5.2.3. Drowsiness and Vigilance Monitoring

Drowsiness and reduced vigilance are major contributors to traffic accidents. Traditional vigilance monitoring approaches, such as facial recognition and steering wheel-based detection, are prone to external interference, whereas EEG signals directly reflect the brain’s arousal level and thus enable higher detection accuracy. Being positioned in the ear canal or behind the ear, ear-EEG devices do not interfere with driving operations, allowing real-time and continuous monitoring of vigilance and drowsiness [122]. In particular, in-ear EEG can effectively discriminate between alert and drowsy states by quantifying power changes in the α and θ frequency bands. Meanwhile, in-ear EEG shows strong anti-interference performance in real-world driving environments, with limited susceptibility to head motion, ambient noise, and lighting variations, supporting reliable in-driving monitoring and early warning of drowsiness and inattention, which is highly valuable for intelligent driving, driver assistance systems, and commercial vehicle safety.

Drowsiness monitoring is critically important across research domains including traffic safety and occupational surveillance. In the field of traffic safety, Hwang et al. [123] pioneered the application of in-ear EEG for alertness–drowsiness classification in simulated driving, achieving an 88.3% accuracy that outperformed conventional scalp EEG (87.0%). Additionally, Hong et al. [124] developed a multimodal wearable device integrating in-ear EEG, PPG, and ECG, verifying that its drowsiness recognition accuracy in virtual driving was comparable to standard scalp EEG.

Extending beyond road transportation to aviation-related occupational surveillance, van Klaren et al. [125] investigated the feasibility of a cEEGrids ear-EEG system for monitoring fatigue and drowsiness in aviation personnel. Their results showed that ear-EEG could partially detect changes in α-band power related to sleep deprivation, although θ-band power exhibited no significant variation, revealing limited performance under mild fatigue states. Nevertheless, the device demonstrated considerable potential as a noninvasive tool for fatigue monitoring, with further optimization and validation under severe fatigue conditions still needed for real-world application. In a long-term monitoring study, Kaveh, R. et al. [18] combined an in-ear EEG system with machine learning algorithms to automatically classify alertness levels over 35 h of continuous recording. Drowsiness was induced by repetitive gaming tasks, with subjective sleepiness rated every 5 min using the KSS. Prominent modulation of α waves (8–12 Hz) was observed during eye closure, and the overall drowsiness detection accuracy reached 93.3%.

Overall, in-ear EEG delivers favorable performance in alertness and fatigue assessment compared with traditional scalp EEG. With its unobtrusive wearing style, high comfort, and capability for real-time monitoring, in-ear EEG serves as a promising technical solution for drowsiness detection in traffic safety and occupational health scenarios.

#### 5.2.4. Emotion, Stress, and Attention Monitoring

Mental state monitoring, including emotion, stress and attention assessment, is a key direction in wearable brain science. Traditional EEG requires multi-channel electrodes, which is not conducive to daily wear. In contrast, in-ear EEG can effectively classify mental states with only single or dual-channel signals, boasting inherent advantages in consumer-grade mental health monitoring.

In the field of emotion recognition, as research continues to advance, Li et al. [126] first confirmed the feasibility of a single-channel, low-cost in-ear EEG device for emotion recognition. Specifically, the device achieved a high 94.1% cross-validation accuracy in binary valence (positive/negative) classification, while its performance in multi-class arousal recognition (covering excited, relaxed, and negative states) remained moderate at 58.8%—a key challenge that underscored the limitations of the early setup. Subsequently, Athavipach et al. [25] further expanded the application of in-ear EEG by developing a wearable device dedicated to emotion monitoring, which could classify four discrete emotions. A pivotal finding from their research is that the classification performance of this in-ear device was not significantly different from that of conventional T7/T8 scalp electrodes, thereby providing strong evidence that ear canal-derived signals are sufficient for accurate emotion discrimination and laying a solid foundation for replacing bulky scalp EEG systems with discreet, daily-usable in-ear devices. In recent years, research has shifted toward a more nuanced understanding of emotions by adopting the valence-arousal dimensional (Circumplex) model, and Arnesano et al. [127] have made notable progress in this regard. They utilized a single-channel in-ear EEG placed at the mastoid region, combined with artificial neural networks (ANN) and explainable AI (XAI) techniques, and conducted experiments on 24 participants. Their study identified β and γ band power as key neurophysiological biomarkers for emotion, achieved a classification accuracy of over 70%, and effectively verified that in-ear EEG is capable of supporting sophisticated, dimension-based emotion recognition while highlighting the distinct neural oscillatory patterns associated with different emotional states.

Regarding stress monitoring, ear-EEG has demonstrated considerable potential as a noninvasive and convenient monitoring tool. Specifically, Ahn et al. [128] developed a headband-type hybrid in-ear EEG system that collects both EEG and ECG signals from behind the ear, achieving an 87.5% accuracy in binary classification of stress and non-stress states—effectively verifying the feasibility of ear-EEG for stress detection. Furthermore, Ha, U et al. [129] advanced this research by integrating in-ear EEG with hemodynamic encephalography (HEG) and heart rate variability (HRV) for mental stress monitoring. Unlike single-modal approaches, this multimodal integration leverages complementary physiological signals, ensuring the stability and robustness of stress monitoring results.

In terms of attention detection, in-ear EEG also exhibits significant application potential in identifying cognitive states. Specifically, Jeong et al. [42,130] employed in-ear EEG combined with an echo state network to effectively recognize the user’s attention state, thereby providing reliable technical support for attention training and cognitive enhancement.

At present, in-ear EEG can realize the recognition of basic emotions such as happiness, anger, sadness, and relaxation, as well as the continuous monitoring of stress and attention, and can be applied to mental health management, learning cognitive monitoring, human–computer interaction, and other fields.

It is worth noting that the included literature adopted varied research cohorts according to respective experimental objectives: most BCI and basic brain state validation tests recruited healthy volunteers [36,90], while epilepsy and dementia-related monitoring investigations enrolled confirmed clinical patients [42,130].

Beyond technical limitations, long-term daily deployment of such wearable in-ear EEG monitoring also faces prominent non-technical barriers from regulation, ethics and data protection perspectives. At present, dedicated medical certification specifications for wearable neuro-monitoring equipment remain incomplete globally, hindering formal clinical approval. Ethically, continuous long-term brain signal collection requires standardized informed consent procedures for enrolled users [36,114]. Meanwhile, massive collected brain and physiological information involves sensitive personal data, lacking mature privacy protection norms to prevent information abuse and leakage, which further restricts large-scale clinical popularization.

Among all validated in-ear EEG applications, overnight home sleep staging and driver drowsiness monitoring stand closest to commercialization and real-world mass deployment, backed by abundant clinical verification, mature algorithm frameworks and ongoing industrial product iterations as summarized in cited literature [18,39,112]. Consumer-grade mental stress and basic emotion monitoring also advance rapidly toward commercial hearable integration, though still constrained by inconsistent individual signal features. By contrast, epilepsy long-term ambulatory screening and all forms of endogenous BCI (motor/speech imagery control) remain confined to laboratory and small-scale clinical trials due to insufficient cross-subject model robustness and strict medical certification thresholds, requiring further multi-center validation before market launch.

To quantitatively summarize the technical indicators and practical performance of existing representative in-ear EEG studies across BCI and brain state monitoring fields, a cross-study quantitative comparison table is supplemented below, including electrode configuration, signal quality, experimental scale and real-world verification information extracted from cited literatures (Table 3).

### 5.3. Developmental Roadmap of In-Ear EEG Systems

**Figure 4:**
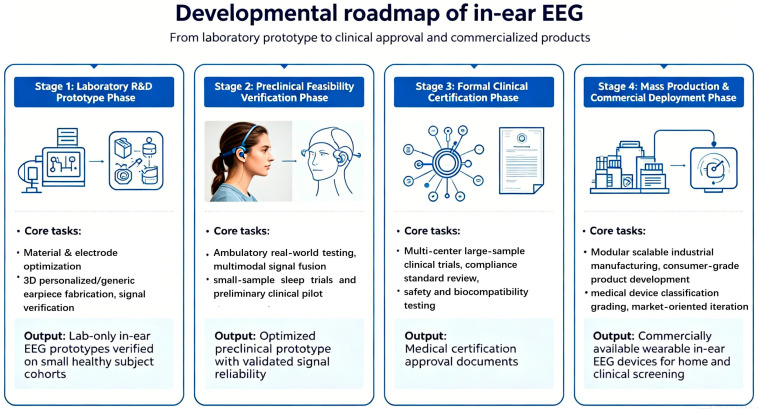
Developmental roadmap of in-ear EEG from laboratory prototype to clinical approval and commercialized products.

Based on the aforementioned research findings and application progress, the whole developmental process of in-ear EEG can be divided into four key stages: laboratory prototype development with basic technical verification, preclinical testing and small-scale clinical trials for performance optimization, multi-center clinical assessment and regulatory certification, and ultimately mass production and commercialization of graded products for home and clinical applications. A developmental roadmap spanning from lab prototype to commercialization is summarized in Figure 4 to intuitively display the full industrial transformation path of in-ear EEG technology.

## 6. Limitations

Despite the considerable promise of in-ear EEG for wearable brain monitoring, this technology still faces critical limitations that hinder its clinical translation and widespread commercial adoption. Nevertheless, targeted and effective solutions have been proposed to alleviate each of these challenges. The contradiction of electrode-skin contact is a major problem. Dry electrodes are comfortable and suitable for long-term wear but have high impedance and unstable contact. Wet electrodes and gold-plated electrodes feature low impedance but reduce wearing comfort and may cause skin irritation. This problem can be solved by developing flexible dry electrodes, hydrogel-based semi-dry electrodes and 3D printed customized electrodes, which balance impedance, biocompatibility and long-term wearing comfort [14,27]. Large anatomical variability is another prominent constraint, as substantial individual differences in ear-canal morphology significantly restrict the standardization of electrode design, placement, and large-scale production; this problem can be systematically mitigated by employing 3D scanning, anthropometric databases, and statistical shape models to develop group-fit, cluster-fit, and fully personalized devices for enhanced anatomical adaptability [40]. Poor ergonomics and wearing stability also severely impede practical performance, because insufficient fitting stability directly degrades both user comfort and signal consistency during long-term monitoring; this issue can be notably improved by adopting adaptive personalized structures such as custom 3D-printed earpieces and self-adjusting electrodes to strengthen fitting stability and optimize electrode–skin contact [2].

Another inherent defect is the limited spatial sampling range and poor signal source localization ability. In-ear electrodes are limited to the ear area and can only collect signals from the temporal lobe, failing to effectively cover the frontal lobe and other remote cortical regions [110]. This defect makes the technology incompetent for assessing frontal lobe-related neural activities and cognitive functions. Furthermore, limited electrode quantity and fixed placement positions greatly weaken the ability to trace and localize the origin of brain signals, so accurate three-dimensional source localization cannot be realized with current in-ear EEG configurations, which becomes a major obstacle for advanced neurological diagnosis and brain mechanism research [9].

There is a fundamental trade-off in the design of in-ear EEG devices: wearing comfort and signal quality cannot be optimized at the same time. To ensure stable electrode contact and high-quality signals, the device needs to fit tightly against the ear canal, which will cause compression pain and reduce comfort during all-day wear [25]. In contrast, overly soft and loose structures improve user experience greatly, but they lead to unstable contact, increased impedance drift and motion artifacts, thus degrading signal quality. This conflicting relationship exists in both wet and dry electrode designs, and it remains a tough challenge to fully reconcile the two requirements for long-term wearable applications [14,25,27].

Another non-negligible drawback of canal-placed in-ear EEG electrodes is the hearing attenuation caused by earplug-like occlusion effect, which is especially prominent for flexible self-fitting electrode earpieces [2,13,49]. Qualitatively, solid or dense flexible electrodes occupy the ear canal cavity and block natural sound propagation, altering users’ normal auditory experience during long-duration wearing and introducing unwanted brain activity fluctuations triggered by degraded hearing [49]. Quantitatively, fully enclosed in-ear electrode structures generally lead to mid-frequency sound attenuation over 10 dB (500–3000 Hz), while thin open-frame electrode designs lower this value to 3–6 dB; such auditory degradation severely compromises the practical reliability of in-ear EEG in attention monitoring, daytime ambulatory epilepsy detection and daily BCI tasks, where intact natural hearing is indispensable to guarantee authentic physiological data recording [8,13].

From the perspective of clinical translation, multiple inherent limitations of in-ear EEG—such as low spatial resolution, unstable electrode-skin contact, individual anatomical variations and motion artifacts—have become major obstacles to large-scale multi-center clinical trials and widespread clinical application [36,51]. Current research is mostly limited to small-scale experiments on healthy volunteers, with a lack of preliminary data from patient cohorts and standardized multi-center datasets for validating diagnostic reliability. Although advanced algorithms can partially compensate for insufficient spatial resolution, the improvement is far from meeting the requirements of formal clinical diagnosis. Meanwhile, inconsistent electrode specifications and testing protocols across studies result in poor experimental repeatability and impede unified clinical validation and regulatory approval [51]. Additionally, personalized and generic earpieces each have obvious drawbacks: customized solutions bring higher costs and inconsistent fitting performance, while generic devices suffer unstable in-vivo contact performance [39,44]. These issues further complicate the design of unified multi-center trials and slow the clinical translation of this technology from laboratory prototypes to practical use.

In addition, conflicting manufacturing and standardization constraints bring extra barriers, since customized earpieces enhance fitness but raise costs and manufacturing complexity, whereas generic designs reduce costs but result in unstable fitting and inconsistent signal quality; Such cost-performance divergence between personalized and generic ear configurations constitutes the core reason for the aforementioned manufacturing tradeoff dilemma. This conflict can be effectively eased by combining scalable manufacturing techniques with modular design to reduce costs while maintaining stable and uniform performance [30]. Moreover, insufficient spatial resolution remains an inherent bottleneck, because the fixed ear location and limited electrode channels lead to intrinsically low spatial resolution, rendering in-ear EEG unsuitable for large-area cortical mapping; this limitation can be partially compensated through multimodal fusion sensing and optimized integrated circuit design. Furthermore, lack of unified standardization obstructs academic comparison and industrialization, because the absence of standardized norms for electrode naming, quantity, placement, and reference configuration leads to inconsistent experimental setups and difficulties in cross-study validation; this obstacle can be relieved by establishing unified electrode coding systems and optimized reference strategies [51]. Meanwhile, poor cross-individual generalization weakens model reliability, since large inter-subject variations in ear anatomy and neural signals reduce the robustness and transferability of classification models; this limitation can be effectively enhanced via transfer learning, federated learning, and ensemble learning to improve cross-user adaptability.

Wearable in-ear EEG is restricted by hardware and power limitations. The confined space of the ear canal constrains battery size, leading to short runtime during multi-channel sampling and real-time algorithm operation [48,51,67]. Additionally, analog circuits and on-board AI modules consume substantial power, further shortening battery life. Real-time onboard AI is only feasible for lightweight analytical tasks under such constraints. Miniaturized chips are also plagued by insufficient computing capability and poor heat dissipation, making it difficult to run complex signal processing algorithms locally [67,79]. To address these challenges that hinder long-term practical use, researchers can adopt ultra-low power chips, edge computing architectures and advanced energy harvesting technologies to improve battery endurance and on-device computational performance under miniaturization requirements [67,79].

## 7. Future Outlook

Looking forward, despite the remarkable progress of in-ear EEG as a wearable brain monitoring technology, its transition from laboratory prototypes to mature clinical and consumer applications remains dependent on systematic and coordinated innovation across key technical and translational dimensions.

### 7.1. Material Innovation: Core Foundation for Performance Breakthrough

High-performance flexible dry electrodes and hydrogel-based semi-dry electrodes will be developed to achieve ultra-low impedance, high biocompatibility and long-term stability. Advanced conductive materials such as conductive polymers, porous metals and flexible composites will be adopted to balance contact impedance, wearing comfort and durability, so as to provide a material foundation for reliable long-term monitoring [14].

### 7.2. Structural Design: Adaptive Personalization and Ergonomic Optimization

Adaptive personalized structures (custom 3D-printed earpieces, self-adjusting electrodes) will enhance fitting stability and electrode–skin contact. Ergonomic designs compatible with diverse ear canal anatomies will be developed to improve wearing stability and comfort, supporting robust and long-duration wearable applications [40].

### 7.3. Hardware and Circuit Integration: Miniaturization and Low-Noise Performance

At the hardware circuit level, near-ear low-noise circuits and highly integrated front-end designs will reduce interference directly at the signal source. Key targets include miniaturization, ultra-low power consumption, and high common-mode rejection, enabling clear and stable signal recording even in complex daily environments [64]. These optimizations will also help expand the scope of real-time onboard AI applications.

### 7.4. Algorithm and System Fusion: Multimodal Intelligence and Robustness

Multimodal fusion sensing (combining in-ear EEG with accelerometers, microphones, PPG, ECG) will enable comprehensive real-time physiological monitoring. Advanced signal processing algorithms will be deployed to suppress motion and physiological artifacts, improve long-term signal stability, and support intelligent health management and human–computer interaction [75]. Multimodal integration combining in-ear EEG with ECG, PPG, EMG and inertial sensors will evolve into an indispensable technical route for future practical wearable monitoring. Fused multi-physiological data compensates the inherent drawbacks of single in-ear EEG including insufficient spatial coverage and heavy muscle/jaw artifacts, improves classification stability under daily dynamic scenarios, and greatly promotes reliable real-world clinical translation of wearable neuro-monitoring equipment [118,120].

### 7.5. Clinical Validation: Functional Verification and Scene Landing

Target clinical applications including sleep monitoring, epilepsy detection, fatigue warning, and emotion recognition will be validated with large-sample real-world data. Long-term safety, effectiveness, and stability will be systematically verified to support translation from laboratory prototypes to clinical and daily-use products [81].

### 7.6. Standardization: Unified Specifications for Industrialization

Uniform standards will be established for electrode placement, channel configuration, data format, performance metrics, and biocompatibility testing. Standardization will improve cross-study comparability, reduce redundant development, and accelerate large-scale commercial deployment of in-ear EEG systems.

### 7.7. Mitigation of Electrode-Induced Hearing Attenuation

To resolve the occlusion-caused hearing decline of in-ear devices, three feasible develop mental routes are proposed [8,49]. First, open-channel hollow earpiece architectures and slim-edged electrode layouts will be developed to reserve inherent acoustic transmission pathway within ear canal and restrict overall sound attenuation under 3 dB [8,49]. Second, porous breathable conductive composite materials will replace dense solid substrates, balancing stable electrode-canal contact and favorable acoustic permeability [5,25]. Third, hybrid hardware schemes combining open in-ear and supplementary around-ear electrodes are recommended for attention monitoring, daytime epilepsy tracking and daily BCI applications; meanwhile, dedicated signal preprocessing algorithms will be refined to eliminate artifacts originating from abnormal brain responses induced by compromised hearing [55,95].

### 7.8. Emerging Advanced Technology-Driven Development

Novel technologies including flexible electronics, self-powered hardware, edge AI and digital twin will power the next iteration of personalized in-ear neuro-monitoring [67,79]. High-performance flexible electronic materials facilitate seamless conformal contact with irregular ear canal tissues and effectively suppress contact-impedance fluctuations induced by body movement [8]. Self-powered designs harvesting body kinetic or thermal energy can shrink device volume and remove frequent battery replacement limits for long-duration wearable monitoring [10]. Compact edge AI algorithms will be embedded into on-ear chips to accomplish local real-time artifact filtering and brain-state analysis without cloud transmission delay [67,79]. Furthermore, digital twin techniques can build personalized digital models of individual ear anatomy and corresponding EEG features, enabling dynamic adaptive optimization of hardware parameters and classification pipelines to achieve patient-tailored long-term neuro-monitoring [40].

## 8. Conclusions

In-ear EEG has emerged as a promising wearable neurotechnology enabling long-term, unobtrusive, and ambulatory brain monitoring outside clinical settings. Advances in flexible electrode materials, personalized 3D-printed structures, miniaturized circuits, and AI-driven signal processing have validated its feasibility in sleep assessment, epilepsy tracking, drowsiness detection, emotion recognition, and BCIs. Moreover, the absence of unified standards for electrode configuration, testing protocols, and clinical validation hinders large-scale translation.

Based on the aforementioned research findings, in-ear EEG is preferentially applicable to scenarios requiring long-term ambulatory home monitoring, neurophysiological signals originating predominantly from the temporal lobe, trend-oriented screening rather than definitive diagnosis, and no demand for high spatial resolution across the entire cerebral cortex. Its prioritized implementations cover lightweight BCIs [81], at-home sleep staging, out-of-hospital surveillance for temporal-lobe epilepsy, as well as real-time detection of drowsiness, emotion and cognitive states [39,44]. Additional feasible uses include auxiliary screening for cognitive impairment and auditory dysfunction alongside the development of consumer-grade wearable electronics [10,26]. Restricted by limited spatial brain coverage and susceptibility to motion artifacts, this technique is unsuitable for high-precision clinical tasks such as whole-brain lesion localization and preoperative pathological diagnosis [51].

This review offers new insights by revealing the inherent trade-offs between electrode performance, ergonomic design and manufacturability. It also assesses the performance of current signal processing and AI algorithms in real-world settings and points out their bottlenecks in cross-user and cross-dataset generalization. We have formulated a phased roadmap for technology translation, which distinguishes the application boundaries and limitations of in-ear EEG from conventional scalp EEG and other wearable EEG devices. Additionally, we address outstanding issues including standardization, clinical certification and data security for future research. Future advances in flexible materials, adaptive structures, multimodal fusion and unified validation will drive the development of in-ear EEG. After systematic optimization, this technology will become a compact and reliable solution for daily neurological monitoring and human–computer interaction.

## Figures and Tables

**Table 1:** Comparison between dry and wet electrodes for in-ear EEG.

Feature	Wet Electrodes	Dry Electrodes
Contact Impedance	Baseline impedance low (<10 kΩ @10 Hz); small fluctuation in short term [14]	Baseline impedance higher (7 kΩ – >100 kΩ@10 Hz); large individual differences and real-time variation [14]
Motion Artifacts	Low susceptibility; conductive medium buffers relative displacement between electrode and ear skin; weak jaw/head movement causes few artifacts [20]	High susceptibility; no adhesive electrolyte for fixation; sliding easily occurs under head rotation, chewing and walking [20]
Signal Quality	High SNR, low baseline drift; reliable for capturing weak brain electrical signals [9]	Lower SNR, more background noise; signal distortion easily occurs under poor contact [9]
Operation & Setup	Complex procedure, long setup time; dependent on trained personnel to apply gel/paste [14,30]	Simple operation, rapid placement; users can self-install without professional assistance [30]
Comfort & Post-Processing	Gel/paste residue inside ear canal; easy to cause itching and skin irritation; cumbersome cleaning [24]	No viscous residue; high wearing comfort; clean and convenient after use [24]
Long-Term Stability	Electrolyte evaporates over time; impedance rises continuously, signal degrades; not suitable for multi-hour continuous recording [27]	No volatile components; stable electrical performance; support repeated use and long-duration monitoring [27]
Reusability	Disposable in most cases; residual electrolyte limits reuse [19]	Good reusability; structural integrity maintained after repeated wearing [29]
Anatomical Adaptability	Good compliance with irregular ear canal skin and different ear shapes	Reliant on physical compression; poor contact for narrow or deformed ear canals [28]
Main Application Fields	Hospital clinical EEG, epilepsy assessment, auditory evoked potential testing	Portable wearable EEG, sleep monitoring, BCI, consumer health equipment [25]

**Table 2:** Performance Comparison of In-Ear EEG with Conventional Scalp EEG.

Metric	In-Ear EEG Hardware	Conventional Scalp EEG Hardware
SNR	Comparable SNR, slightly lower amplitude (10–20 dB) [9]	Higher SNR, gold-standard signal fidelity [51]
Contact Impedance	Higher and more variable (dry electrodes: ~7–133 kΩ; wet: <10 kΩ) [13]	Low and stable (<10 kΩ for Ag/AgCl paste electrodes)
Motion Artifacts	More sensitive to jaw/movement artifacts; less affected by eye blinks [58]	Less sensitive to body motion; affected by eye/head movement [58]
Wearability & Discreteness	Ultra-wearable, invisible, no head restriction [3]	Bulky, obtrusive, requires electrode cap
Setup & Preparation Time	Fast (seconds–minutes), no gel/paste, self-applicable [30]	Long (20–60 min), needs gel/paste, professional setup [30]
Hair Interference	No interference (ear canal is hairless) [2]	Severe interference; requires hair parting/gel [2]
Long-Term Monitoring Stability	Excellent for 24 h+ ambulatory/home use [39]	Poor; gel dries, impedance rises, discomfort [2]
Spatial Coverage & Resolution	Limited (temporal lobe focus), low spatial resolution [29]	Full brain coverage (10–20 system), high spatial resolution [51]
Device Customization	Personalized (molded earpieces) [1] or generic (foam tips) [21]	Standardized caps; limited customization [2]
Clinical Diagnostic Suitability	Limited to screening/ambulatory monitoring	Gold-standard for epilepsy, sleep, neurological diagnosis

**Table 3:** Performance comparison of in-ear EEG systems.

Study	Number of Channels	Electrode (Dry/Wet)	Electrode Material	Signal Quality	Core Acquired Signal	Classification Accuracy	Monitoring Duration	Sample Size (Subjects)	Real-World Validation
Looney et al. [110]	3	Wet	AgCl	Slightly lower SNR than scalp EEG	Alpha Wave	N2/N3 κ = 0.65; N1/W κ = 0.60	45-min nap recording	4	Small-sample lab test
Kappel et al. [37]	6	Dry	Ti/IrO_2_	On par with scalp EEG	ASSR, SSVEP, MMN, Alpha Wave	No Classification Metrics	Short-duration recording	12	Small-sample lab test
Kaveh et al. [46]	6	Dry	Silver-Plated Polycarbonate	average SNR: 5.94 dB	ASSR, Alpha Wave	No Classification Metrics	Intermittent tests over 3 months	3	lab validation for BCI
Goverdovsky et al. [66]	2	Dry	Silver-Plated Nylon	~10dB	Alpha Wave, ASSR, SSVEP	No Classification Metrics	Short segmented recordings	5	Small-sample lab test
Lee et al. [5]	1	Dry	CNT/PDMS	Moderate, Stable signal	ASSR, N100, AEP, Alpha Wave, SSVEP	No Classification Metrics	Short segmented recordings	6	Laboratory-only validation.
Zhao et al. [86]	4	Wet	Hydrogel + Ag/AgCl	Low impedance, high SNR	SSVEP	No Classification Metrics	short segmented trials	12	Lab-based SSVEP BCI test
Wang et al. [8]	5	Dry/Wet	Gold Wire, Flexible Bioelectronics	Stable impedance, high SNR	SSVEP	No Classification Metrics	Segmented auditory recording	14	Lab-only BCI tests
Ahn et al. [90]	3	Wet	Gold-Plated Electrode	Low noise floor	SSVEP	SSVEP Classification Accuracy: 79.9% ± 13.1%	10 s per SSVEP trial	6	lab-only offline BCI test
Hwang et al. [123]	1	Wet	Metal Electrode	Not Reported	Alpha Wave, Arousal/Drowsiness	Alert-Drowsiness Classification Accuracy: 88.3%	Dynamic short-term recording	13	Yes (Simulated Real Driving Scenario)
Merrill et al. [35]	3	Wet	AgCl	No explicit SNR; impedance < 10 kΩ	Alpha Wave	Thought Authentication Classification Accuracy: 85.4%	Segmented short recording	7	Lab-only test
Li et al. [126]	1	Wet	hydrogel	Moderate signal	Emotion-Related EEG	Emotion Binary Classification Accuracy: 94.1%	Short segmented recordings	12	Lab-only emotion test;
Arnesano et al. [127]	1	Wet	AgCl	No explicit SNR	Emotion-Related EEG	Dimensional Emotion Classification Accuracy >70%	Short segmented recording	24	lab-only proof-of-concept test
Ahn et al. [128]	4	Wet	hydrogel	Ultra-low noise	Stress-Related EEG	Stress Binary Classification Accuracy: 87.5%	10 min stabilization + 6 min per stage	14	Lab feasibility test
Tabar et al. [44]	6	Dry	IrO_2_	10.2% artifact rejection	Full-Band Sleep EEG	Sleep Staging κ = 0.71	Multi-Night At-Home Monitoring	10	At-Home Sleep Monitoring
Borges et al. [112]	1	Dry	IrO_2_	No explicit SNR	Sleep Spindle, K-Complex PSG Synchronous Acquisition	Sleep Staging κ > 0.80	Long-Term At-Home Monitoring	28	At-Home Sleep Assessment
Joyner et al. [115]	4	Wet	Wet electrode with conductive paste	Low false detection rate	Epileptic Seizure Wave, EMG	Epileptic Seizure High Consistency with Scalp EEG	Long-term continuous monitoring	20 Patients with epilepsy	Clinical validation in hospital

## Data Availability

No new data were created or analyzed in this study. Data sharing is not applicable to this article.

## Abbreviations

The following abbreviations are used in this manuscript:

ACT	Actigraphy
AI	Artificial Intelligence
ANN	Artificial Neural Networks
ASSR	Auditory Steady-State Response
BCI	Brain–Computer Interface
BLE	Bluetooth Low Energy
CCA	Canonical Correlation Analysis
CNTP/PDMS	Carbon Nanotube Porins/Polydimethylsiloxane
CNN	Convolutional Neural Network
DC	Direct Current
DPAE	Dual Pathway Autoencoders
ECR	Error Correction Regression
EMD	Empirical Mode Decomposition
ERP	Event-Related Potential
ERD	Event-Related Desynchronization
HEG	Hemodynamic Encephalography
ICA	Independent Component Analysis
IrO_2_	Iridium Dioxide
KSS	Karolinska Sleepiness Scale
LSTM	Long Short-Term Memory
MESD	Multi-layer Ear-Scalp Distillation
MI	Motor Imagery
ML	Machine Learning
NREM	Non-Rapid Eye Movement
PDMS	Polydimethylsiloxane
PSG	Polysomnography
REM	Rapid Eye Movement
RF	Random Forest
Ag/AgCl	Silver/Silver Chloride
SI	Speech Imagery
SNR	Signal-to-Noise Ratio
SSVEP	Steady-State Visual Evoked Potential
STFT	Short-Time Fourier Transform
U-Net	U-shaped Network
VI	Visual Imagery
XAI	Explainable Artificial Intelligence

## References

[1] Looney D., Park C., Kidmose P., Rank M.L., Ungstrup M., Rosenkranz K., Mandic D.P. (2011). An in-the-ear platform for recording electroencephalogram. 2011 Annual International Conference of the IEEE Engineering in Medicine and Biology Society.

[2] Mihai A.Ş., Geman O., Toderean R. Innovative approaches to EEG in the ear: A review. Proceedings of the E-Health and Bioengineering Conference (EHB).

[3] Looney D., Park C., Kidmose P. (2011). In-ear EEG: A wearable platform for real-time brain-monitoring. IEEE Trans. Biomed. Eng..

[4] Kancaoğlu M., Kuntalp M. (2024). Low-cost, mobile EEG hardware for SSVEP applications. HardwareX.

[5] Lee J.H., Lee S.M., Byeon H.J., Hong J.S., Park K.S., Lee S.H. (2014). CNT/PDMS-based canal-typed ear electrodes for inconspicuous EEG recording. J. Neural Eng..

[6] Leung J., Holanda L.J., Wheeler L., Chau T. (2026). Wireless in-ear EEG system for auditory brain-computer interface applications in adolescents. Biomed. Phys. Eng. Express.

[7] Hyung W., Kim M., Kim Y., Im C.H. (2025). DeepAttNet: Deep neural network incorporating cross-attention mechanism for subject-independent mental stress detection in passive brain-computer interfaces using bilateral ear-EEG. Front. Hum. Neurosci..

[8] Wang Z., Shi N., Zhang Y., Zheng N., Li H., Jiao Y., Cheng J., Wang Y., Zhang X., Chen Y. (2023). Conformal in-ear bioelectronics for visual and auditory brain-computer interfaces. Nat. Commun..

[9] Moumane H., Pazuelo J., Nassar M., Juez J.Y., Valderrama M., Le Van Quyen M. (2024). Signal quality evaluation of an in-ear EEG device in comparison to a conventional cap system. Front. Neurosci..

[10] Yu L., Herbozo Contreras L.F., Huang Z., Yang Y., Chen B., Kavehei O. (2025). Hearables: Bioelectronics technological challenges and opportunities. Wearable Technol..

[11] De Fazio R., Yalçınkaya Ş.E., Cascella I., Del-Valle-Soto C., De Vittorio M., Visconti P. (2025). Forehead and in-ear EEG acquisition and processing: Biomarker analysis and memory-efficient deep learning algorithm for sleep staging with optimized feature dimensionality. Sensors.

[12] Lehnen J., Venkatesh P., Yao Z., Aziz A., Nguyen P.V.P., Harvey J., Alick-Lindstrom S., Doyle A., Podkorytova I., Perven G. (2025). Real-time seizure detection using behind-the-ear wearable system. J. Clin. Neurophysiol..

[13] Looney D., Kidmose P., Park C., Ungstrup M., Rank M., Rosenkranz K., Mandic D. (2012). The in-the-ear recording concept: User-centered and wearable brain monitoring. IEEE Pulse.

[14] Petrossian G., Kateb P., Miquet-Westphal F., Cicoira F. (2023). Advances in electrode materials for scalp, forehead, and ear EEG: A mini-review. ACS Appl. Bio Mater..

[15] van der Heijden P., Gilbert C., Jafari S., Lucchini M.A. (2024). Multi-channel soft dry electrodes for electrocardiography acquisition in the ear region. Sensors.

[16] Wang Z., Zhao N., Shen G., Jiang C., Liu J. (2023). MEMS-based flexible wearable tri-polar concentric ring electrode array with self-adhesive graphene gel for EEG monitoring. IEEE Sens. J..

[17] Umar A.H., Othman M.A., Che Harun F.K., Yusof Y. (2021). Dielectrics for non-contact ECG bioelectrodes: A review. IEEE Sens. J..

[18] Kaveh R., Schwendeman C., Pu L., Arias A.C., Muller R. (2024). Wireless ear EEG to monitor drowsiness. Nat. Commun..

[19] Singh K., Lin C.-C., Huang W.-H., Lei W.-L., Chiueh H., Wang Y.-H., Chang P.-H., Lin R.-Z., Huang W.-C. (2025). Ultrabioconformal, self-healable, and antioxidized polydopamine-inspired nanowire hydrogels enable resolving power in forehead and ear electroencephalograms for brain function assessment. ACS Appl. Mater. Interfaces.

[20] Liu Q., Yang L., Zhang Z., Yang H., Zhang Y., Wu J. (2023). The feature, performance, and prospect of advanced electrodes for electroencephalogram. Biosensors.

[21] Xiong Z., Qiang L., Kilsgaard S., Moradi F., Kappel S.L., Kidmose P. (2016). A wearable ear-EEG recording system based on dry-contact active electrodes. 2016 IEEE Symposium on Vlsi Circuits (Vlsi-Circuits).

[22] Kawana T., Zemba Y., Ichikawa R., Miki N. (2023). Easily attach/detach reattachable EEG headset with candle-like microneedle electrodes. Micromachines.

[23] Eickenscheidt M., Schäfer P., Baslan Y., Schwarz C., Stieglitz T. (2020). Highly porous platinum electrodes for dry ear-EEG measurements. Sensors.

[24] Kappel S.L., Kidmose P. (2022). Characterization of dry-contact EEG electrodes and an empirical comparison of Ag/AgCl and IrO_2_ electrodes. 2022 44th Annual International Conference of the IEEE Engineering in Medicine & Biology Society (EMBC).

[25] Athavipach C., Pan-ngum S., Israsena P. (2019). A wearable in-ear EEG device for emotion monitoring. Sensors.

[26] Bertelsen A.R., Bladt H., Christensen C.B., Kappel S.L., Toft H.O., Rank M.L., Mikkelsen K.B., Kidmose P. (2019). Generic dry-contact ear-EEG. 2019 41st Annual International Conference of the IEEE Engineering in Medicine and Biology Society (EMBC).

[27] Walsh A., Shanker S., Valdes A.L. (2025). Classifying neurodegenerative diseases from selected temporal EEG electrodes: Towards ear-EEG applications. 2025 47th Annual International Conference of the IEEE Engineering in Medicine and Biology Society (EMBC).

[28] Lee M.S., Paul A., Joung T.H., Xu Y., Wu J., Hairston W.D., Cauwenberghs G. (2023). Scalable anatomically-tunable fully in-ear dry-electrode array for user-generic unobtrusive electrophysiology. 2023 45th Annual International Conference of the IEEE Engineering in Medicine & Biology Society (EMBC).

[29] Meiser A., Knoll A.L., Bleichner M.G. (2024). High-density ear-EEG for understanding ear-centered EEG. J. Neural Eng..

[30] He X., Liang H., Li H., Liu R., Wang Y. (2025). Design and performance evaluation of a real-time single-channel ear-EEG acquisition system for wearable applications. 2025 47th Annual International Conference of the IEEE Engineering in Medicine and Biology Society (EMBC).

[31] Borges H.B., Zaar J., Alickovic E., Christensen C.B., Kidmose P. (2025). The speech reception threshold can be estimated using EEG electrodes in and around the ear. J. Neural Eng..

[32] Hinrichs H., Scholz M., Baum A.K., Kam J.W.Y., Knight R.T., Heinze H.-J. (2020). Comparison between a wireless dry electrode EEG system with a conventional wired wet electrode EEG system for clinical applications. Sci. Rep..

[33] Zibrandtsen I., Kidmose P., Otto M., Ibsen J., Kjaer T.W. (2016). Case comparison of sleep features from ear-EEG and scalp-EEG. Sleep Sci..

[34] Christensen C.B., Harte J.M., Lunner T., Kidmose P. (2018). Ear-EEG-based objective hearing threshold estimation evaluated on normal hearing subjects. IEEE Trans. Biomed. Eng..

[35] Merrill N., Curran M.T., Gandhi S., Chuang J. (2019). One-step, three-factor passthought authentication with custom-fit, in-ear EEG. Front. Neurosci..

[36] Musaeus C.S., Waldemar G., Andersen B.B., Høgh P., Kidmose P., Hemmsen M.C., Rank M.L., Kjær T.W., Frederiksen K.S. (2022). Long-term EEG monitoring in patients with Alzheimer’s disease using ear-EEG: A feasibility study. J. Alzheimer’s Dis..

[37] Kappel S.L., Rank M.L., Toft H.O., Andersen M., Kidmose P. (2019). Dry-contact electrode ear-EEG. IEEE Trans. Biomed. Eng..

[38] Das A., Basu S., Adarsh A., Gubbi J., Muralidharan K., Meghana S., Mahendiran S., Biradar A., Pradhan U., Chakravarty T. (2022). Surface potential simulation and electrode design for in-ear EEG measurement. 2022 44th Annual International Conference of the IEEE Engineering in Medicine & Biology Society (EMBC).

[39] Tabar Y.R., Mikkelsen K.B., Rank M.L., Hemmsen M.C., Otto M., Kidmose P. (2021). Ear-EEG for sleep assessment: A comparison with actigraphy and PSG. Sleep Breath..

[40] Yu L., Xu Z., Contreras L.H., Kavehei O. (2024). An additively manufactured 3D printed electronics system for personalized ear-EEG. 2024 International Conference on Electrical, Computer and Energy Technologies (ICECET).

[41] Kappel S.L., Kidmose P. (2017). High-density ear-EEG. 2017 39th Annual International Conference of the IEEE Engineering in Medicine and Biology Society (EMBC).

[42] Jeong D.-H., Jeong J. (2020). In-ear EEG based attention state classification using echo state network. Brain Sci..

[43] Mikkelsen K.B., Kappel S.L., Mandic D.P., Kidmose P. (2015). EEG recorded from the ear: Characterizing the ear-EEG method. Front. Neurosci..

[44] Tabar Y.R., Mikkelsen K.B., Shenton N., Kappel S.L., Bertelsen A.R., Nikbakht R., Toft H.O., Henriksen C.H., Hemmsen M.C., Rank M.L. (2023). At-home sleep monitoring using generic ear-EEG. Front. Neurosci..

[45] Kidmose P., Looney D., Jochumsen L., Mandic D.P. (2013). Ear-EEG from generic earpieces: A feasibility study. 2013 35th Annual International Conference of the IEEE Engineering in Medicine and Biology Society (EMBC).

[46] Kaveh R., Doong J., Zhou A., Schwendeman C., Gopalan K., Burghardt F.L., Arias A.C., Maharbiz M.M., Muller R. (2020). Wireless user-generic ear EEG. IEEE Trans. Biomed. Circuits Syst..

[47] Schwendeman C., Kaveh R., Muller R. (2022). Drowsiness detection with wireless, user-generic, dry electrode ear EEG. 2022 44th Annual International Conference of the IEEE Engineering in Medicine & Biology Society (EMBC).

[48] Paul A., Lee M.S., Xu Y., Deiss S.R., Cauwenberghs G. (2023). A versatile in-ear biosensing system and body-area network for unobtrusive continuous health monitoring. IEEE Trans. Biomed. Circuits Syst..

[49] Goverdovsky V., von Rosenberg W., Nakamura T., Looney D., Sharp D.J., Papavassiliou C., Morrell M.J., Mandic D.P. (2017). Hearables: Multimodal physiological in-ear sensing. Sci. Rep..

[50] Liang H., Wang Y., Li H., Wang Y., Liu P.X., Liu R. (2023). Development and characterization of a dry ear-EEG sensor with a generic flexible earpiece. IEEE Trans. Instrum. Meas..

[51] Ungureanu A.S.M., Geman O., Toderean R., Miron L., SharghiLavan S. (2025). The next frontier in brain monitoring: A comprehensive look at in-ear EEG electrodes and their applications. Sensors.

[52] Ulate-Campos A., Loddenkemper T. (2024). Review on the current long-term, limited lead electroencephalograms. Epilepsy Behav..

[53] Paul A., Akinin A., Lee M.S., Kleffner M., Deiss S.R., Cauwenberghs G. (2019). Integrated In-Ear Device for Auditory Health Assessment. 2019 41st Annual International Conference of the IEEE Engineering in Medicine and Biology Society (EMBC).

[54] Paul A., Deiss S.R., Tourtelotte D., Kleffner M., Zhang T., Cauwenberghs G. (2019). Electrode-Skin Impedance Characterization of In-Ear Electrophysiology Accounting for Cerumen and Electrodermal Response. 2019 9th International IEEE/EMBS Conference on Neural Engineering (NER).

[55] Kidmose P., Looney D., Mandic D.P. (2012). Auditory Evoked Responses from Ear-EEG Recordings. 2012 Annual International Conference of the IEEE Engineering in Medicine and Biology Society.

[56] Meiser A., Tadel F., Debener S., Bleichner M.G. (2020). The sensitivity of ear-EEG: Evaluating the source-sensor relationship using forward modeling. Brain Topogr..

[57] Geirnaert S., Kappel S.L., Kidmose P. (2025). A direct comparison of simultaneously recorded scalp, around-ear and in-ear EEG for neural selective auditory attention decoding to speech. Sci. Rep..

[58] He Y. (2025). Comparative analysis of ear electroencephalography (ear-EEG) and scalp electroencephalography (scalp-EEG) in wearable brain-computer interfaces. Front. Sci. Eng..

[59] Lebiecka-Johansen P., Strom J., Mikkelsen K.B., Cabrera A.F., Madsen R.E., Christensen J.A.E., Hemmsen M.C., Kidmose P. (2025). Benefits of different strategies to adapt sleep scoring models from scalp- to ear-EEG. 2025 47th Annual International Conference of the IEEE Engineering in Medicine and Biology Society (EMBC).

[60] Mandekar S., Holland A., Thielen M., Behbahani M., Melnykowycz M. (2022). Advancing towards ubiquitous EEG, correlation of in-ear EEG with forehead EEG. Sensors.

[61] Crétot-Richter G., De Vos M., Debener S., Bleichner M.G., Voix J. (2023). Assessing focus through ear-EEG: A comparative study between conventional cap EEG and mobile in- and around-the-ear EEG systems. Front. Neurosci..

[62] Mikkelsen K.B., Tabar Y.R., Kappel S.L., Christensen C.B., Toft H.O., Hemmsen M.C., Rank M.L., Otto M., Kidmose P. (2019). Accurate whole-night sleep monitoring with dry-contact ear-EEG. Sci. Rep..

[63] Kappel S.L., Looney D., Mandic D.P., Kidmose P. (2017). Physiological artifacts in scalp EEG and ear-EEG. Biomed. Eng. Online.

[64] Jiang X., Bian G.B., Tian Z. (2019). Removal of Artifacts from EEG Signals: A Review. Sensors.

[65] Kidmose P., Looney D., Ungstrup M., Rank M.L., Mandic D.P. (2013). A study of evoked potentials from ear-EEG. IEEE Trans. Biomed. Eng..

[66] Goverdovsky V., Looney D., Kidmose P., Mandic D.P. (2016). In-ear EEG from viscoelastic generic earpieces: Robust and unobtrusive 24/7 monitoring. IEEE Sens. J..

[67] Lee J., Lee K.-R., Ha U., Kim J.-H., Lee K., Gweon S. (2019). A 0.8-V 82.9-μW in-ear BCI controller IC with 8.8 PEF EEG instrumentation amplifier and wireless BAN transceiver. IEEE J. Solid-State Circuits.

[68] Chen X., Xu X., Liu A., McKeown M.J., Wang Z.J. (2018). The use of multivariate EMD and CCA for denoising muscle artifacts from few-channel EEG recordings. IEEE Trans. Instrum. Meas..

[69] Chen X., Peng H., Yu F.Q., Wang K. (2017). Independent vector analysis applied to remove muscle artifacts in EEG data. IEEE Trans. Instrum. Meas..

[70] Safieddine D., Kachenoura A., Albera L., Birot G., Karfoul A., Pasnicu A., Biraben A., Wendling F., Senhadji L., Merlet I. (2012). Removal of muscle artifact from EEG data: Comparison between stochastic (ICA and CCA) and deterministic (EMD and wavelet-based) approaches. EURASIP J. Adv. Signal Process..

[71] Mai N.-D., Nguyen H.-T., Chung W.-Y. (2023). Real-time on-chip machine-learning-based wearable behind-the-ear electroencephalogram device for emotion recognition. IEEE Access.

[72] Zambrana-Vinaroz D., Vicente-Samper J.M., Manrique-Cordoba J., Sabater-Navarro J.M. (2022). wearable epileptic seizure prediction system based on machine learning techniques using ECG, PPG and EEG signals. Sensors.

[73] Wang B., Deng F., Jiang P. (2024). EEGDiR: Electroencephalogram denoising network for temporal information storage and global modeling through Retentive Network. Comput. Biol. Med..

[74] Porr B., Daryanavard S., Muñoz Bohollo L., Cowan H., Dahiya R. (2022). Real-time noise cancellation with deep learning. PLoS ONE.

[75] Roy Y., Banville H., Albuquerque I., Gramfort A., Falk T.H., Faubert J. (2019). Deep learning-based electroencephalography analysis: A systematic review. J. Neural Eng..

[76] Cai Y., Meng Z., Huang D. (2025). DHCT-GAN: Improving EEG Signal Quality with a Dual-Branch Hybrid CNN-Transformer Network. Sensors.

[77] Pawan, Dhiman R. (2023). Machine Learning Techniques for Electroencephalogram Based Brain-Computer Interface: A Systematic Literature Review. Meas. Sens..

[78] Devi B.A. (2026). deep learning-based epileptic seizure detection from EEG signals and PPG signals using LSTM and CNN models. Int. J. Neurosci..

[79] Zhu L.Q., Wang C.M., He Z.H., Zhang Y. (2022). A lightweight automatic sleep staging method for children using single-channel EEG based on edge artificial intelligence. World Wide Web.

[80] Almanza J.C.N., Ota L., Horie K., Kawana F., Kokubo T., Yanagisawa M., Kitagawa H. (2025). Applicability of LSTM and double decoders on U-Net models for sleep and arousal scoring using in-home EEG signals. 2025 47th Annual International Conference of the IEEE Engineering in Medicine and Biology Society (EMBC).

[81] Zhang J., Li J., Huang Z., Huang D., Yu H., Li Z. (2023). Recent progress in wearable brain–computer interface (BCI) devices based on electroencephalogram (EEG) for medical applications: A review. Health Data Sci..

[82] Lee Y.-E., Shin G.-H., Lee M., Lee S.-W. (2021). Mobile BCI dataset of scalp- and ear-EEGs with ERP and SSVEP paradigms while standing, walking, and running. Sci. Data.

[83] Brenner C.A., Krishnan G.P., Vohs J.L., Ahn W.Y., Hetrick W.P., Morzorati S.L., O’Donnell B.F. (2009). Steady state responses: Electrophysiological assessment of sensory function in schizophrenia. Schizophr. Bull..

[84] Sergeeva A., Christensen C.B., Kidmose P. (2022). Investigation of the effect of spatial filtering for detecting auditory steady-state responses recorded from ear-EEG. 2022 44th Annual International Conference of the IEEE Engineering in Medicine & Biology Society (EMBC).

[85] Sergeeva A., Christensen C.B., Kidmose P. (2024). Effect of stimulus bandwidth on the auditory steady-state response in scalp- and ear-EEG. Ear Hear..

[86] Zhao H., Zheng L., Yuan M., Wang Y., Gao X., Liu R., Pei W. (2023). Optimization of ear electrodes for SSVEP-based BCI. J. Neural Eng..

[87] Liang L., Bin G., Chen X., Wang Y., Gao S., Gao X. (2021). Optimizing a left and right visual field biphasic stimulation paradigm for SSVEP-based BCIs with hairless region behind the ear. J. Neural Eng..

[88] Athavipach C., Pan-ngum S., Israsena P. (2018). Development of low-cost in-the-ear EEG prototype. 2018 15th International Joint Conference on Computer Science and Software Engineering (JCSSE).

[89] Guermandi M., Benatti S., Morinigo V.J.K., Bertini L. (2018). A wearable device for minimally-invasive behind-the-ear EEG and evoked potentials. 2018 IEEE Biomedical Circuits and Systems Conference (BioCAS).

[90] Ahn J.W., Ku Y., Kim D.Y., Sohn J., Kim J.H., Kim H.C. (2018). Wearable in-the-ear EEG system for SSVEP-based brain–computer interface. Electron. Lett..

[91] Liang H., Liu R. (2022). A new generic single-channel ear-EEG recording platform. Proceedings.

[92] Sun Y., Zhang F., Li Z., Liu X., Zheng D., Zhang S., Fan S., Wu X. (2024). Multi-layer ear-scalp distillation framework for ear-EEG classification enhancement. J. Neural Eng..

[93] Eidel M., Pfeiffer M., Ziebell P., Kübler A. (2024). Recording the tactile P300 with the cEEGrid for potential use in a brain-computer interface. Front. Hum. Neurosci..

[94] Debener S., Emkes R., De Vos M., Bleichner M. (2015). Unobtrusive ambulatory EEG using a smartphone and flexible printed electrodes around the ear. Sci. Rep..

[95] Bleichner M.G., Lundbeck M., Selisky M., Minow F., Jäger M., Emkes R., Debener S., De Vos M. (2015). Exploring miniaturized EEG electrodes for brain-computer interfaces: An EEG you do not see?. Physiol. Rep..

[96] Kaongoen N., Jo S. (2017). A novel hybrid auditory BCI paradigm combining ASSR and P300. J. Neurosci. Methods.

[97] Zander T.O., Brönstrup J., Lorenz R., Krol L.R. (2014). Towards BCI-based implicit control in human–computer interaction. Advances in Physiological Computing.

[98] Merrill N., Curran M.T., Yang J.K., Chuang J. (2016). Classifying mental gestures with in-ear EEG. 2016 IEEE 13th International Conference on Wearable and Implantable Body Sensor Networks (BSN).

[99] Palumbo A., Gramigna V., Calabrese B., Ielpo N. (2021). Motor-imagery EEG-based BCIs in wheelchair movement and control: A systematic literature review. Sensors.

[100] Saibene A., Caglioni M., Corchs S., Gasparini F. (2023). EEG-based BCIs on motor imagery paradigm using wearable technologies: A systematic review. Sensors.

[101] Miladinovic A., Ajcevic M., Busan P., Jarmolowska J., Silveri G., Deodato M., Mezzarobba S., Battaglini P.P., Accardo A. (2020). Evaluation of motor imagery-based BCI methods in neurorehabilitation of Parkinson’s disease patients. 2020 42nd Annual International Conference of the IEEE Engineering in Medicine & Biology Society (EMBC).

[102] Uyanik C., Khan M.A., Brunner I.C., Hansen J.P., Puthusserypady S. (2022). Machine learning for motor imagery wrist dorsiflexion prediction in brain-computer interface assisted stroke rehabilitation. 2022 44th Annual International Conference of the IEEE Engineering in Medicine & Biology Society (EMBC).

[103] Kim Y.J., Kwak N.S., Lee S.W. (2018). Classification of motor imagery for Ear-EEG based brain-computer interface. 2018 6th International Conference on Brain-Computer Interface (BCI).

[104] Wu X., Zhang W., Fu Z., Cheung R.T.H., Chan R.H.M. (2020). An investigation of in-ear sensing for motor task classification. J. Neural Eng..

[105] Kaongoen N., Choi J., Jo S. (2021). Speech-imagery-based brain-computer interface system using ear-EEG. J. Neural Eng..

[106] Kaongoen N., Choi J., Jo S. (2022). A novel online BCI system using speech imagery and ear-EEG for home appliances control. Comput. Methods Programs Biomed..

[107] Kilmarx J., Tashev I., Millan J.D.R., Sulzer J., Lewis-Peacock J. (2024). Evaluating the feasibility of visual imagery for an EEG-based brain-computer interface. IEEE Trans. Neural Syst. Rehabil. Eng..

[108] Kuatsjah E., Zhang X., Khoshnam M., Menon C. (2019). Two-channel in-ear EEG system for detection of visuomotor tracking state: A preliminary study. Med. Eng. Phys..

[109] Todoroki S., Phunruangsakao C., Goto K., Kutsuzawa K., Owaki D., Hayashibe M. (2025). Deep learning-based decoding and feature visualization of motor imagery speeds from EEG signals. IEEE Open J. Eng. Med. Biol..

[110] Looney D., Goverdovsky V., Rosenzweig I., Morrell M.J., Mandic D.P. (2016). Wearable In-Ear Encephalography Sensor for Monitoring Sleep: Preliminary Observations from Nap Studies. Ann. Am. Thorac. Soc..

[111] Palo G., Fiorillo L., Monachino G., Bechny M., Wälti M., Meier E., Pentimalli Biscaretti di Ruffia F., Melnykowycz M., Tzovara A., Agostini V. (2024). Comparison analysis between standard polysomnographic data and in-ear-electroencephalography signals: A preliminary study. Sleep Adv..

[112] Borges D.F., Soares J.I., Silva H., Felgueiras J., Batista C., Ferreira S., Rocha N.B., Leal A. (2025). A custom-built single-channel in-ear electroencephalography sensor for sleep phase detection: An interdependent solution for at-home sleep studies. J. Sleep Res..

[113] Hestermann E., Schreve K., Vandenheever D. (2024). Enhancing deep sleep induction through a wireless in-ear EEG device delivering binaural beats and ASMR: A proof-of-concept study. Sensors.

[114] Zibrandtsen I.C., Kidmose P., Christensen C.B., Kjaer T.W. (2017). Ear-EEG detects ictal and interictal abnormalities in focal and generalized epilepsy: A comparison with scalp EEG monitoring. Clin. Neurophysiol..

[115] Joyner M.G., Hsu S.-H., Martin S., Dwyer J., Chen D.F., Sameni R., Waters S.H., Borodin K., Clifford G.D., Levey A.I. (2024). Using a standalone ear-EEG device for focal-onset seizure detection. Bioelectron. Med..

[116] Zeydabadinezhad M., Jowers J., Buhl D., Cabaniss B., Mahmoudi B. (2024). A personalized earbud for non-invasive long-term EEG monitoring. J. Neural Eng..

[117] Vandecasteele K., De Cooman T., Chatzichristos C., Cleeren E., Swinnen L., Macea Ortiz J., Van Huffel S., Dümpelmann M., Schulze-Bonhage A., De Vos M. (2021). The power of ECG in multimodal patient-specific seizure monitoring: Added value to an EEG-based detector using limited channels. Epilepsia.

[118] Nielsen J.M., Zibrandtsen I.C., Masulli P., Sørensen T.L., Andersen T.S., Kjær T.W. (2022). Towards a wearable multi-modal seizure detection system in epilepsy: A pilot study. Clin. Neurophysiol..

[119] Bhagubai M., Vandecasteele K., Swinnen L., Macea J., Chatzichristos C., De Vos M., Van Paesschen W. (2023). The power of ECG in semi-automated seizure detection in addition to two-channel behind-the-ear EEG. Bioengineering.

[120] Zhang J., Swinnen L., Chatzichristos C., Broux V., Proost R., Jansen K., Mahler B., Zabler N., Epitashvilli N., Dümpelmann M. (2024). Multimodal wearable EEG, EMG and accelerometry measurements improve the accuracy of tonic-clonic seizure detection. Physiol. Meas..

[121] Zibrandtsen I.C., Kidmose P., Kjaer T.W. (2018). Detection of generalized tonic-clonic seizures from ear-EEG based on EMG analysis. Seizure.

[122] Gangadharan K.S., Vinod A.P. (2022). Drowsiness detection using portable wireless EEG. Comput. Methods Programs Biomed..

[123] Hwang T., Kim M., Hong S., Park K.S. (2016). Driver drowsiness detection using the in-ear EEG. 2016 38th Annual International Conference of the IEEE Engineering in Medicine and Biology Society (EMBC).

[124] Hong S., Kwon H., Choi S.H., Park K.S. (2018). Intelligent System for Drowsiness Recognition based on Ear Canal Electroencephalography with Photoplethysmography and Electrocardiography. Inf. Sci..

[125] van Klaren C., Maij A., Marsman L., van Drongelen A. (2024). The evaluation of cEEGrids for fatigue detection in aviation. Sleep Adv..

[126] Li G., Zhang Z., Wang G. (2017). Emotion recognition based on low-cost in-ear EEG. 2017 IEEE Biomedical Circuits and Systems Conference (BioCAS).

[127] Arnesano M., Arpaia P., Balatti S., Cosoli G., De Luca M., Gargiulo L., Moccaldi N., Pollastro A., Zanto T., Forenza A. (2026). Development of a measurement procedure for emotional states detection based on single-channel ear-EEG: A proof-of-concept study. Sensors.

[128] Ahn J.W., Ku Y., Kim H.C. (2019). A Novel Wearable EEG and ECG Recording System for Stress Assessment. Sensors.

[129] Ha U., Kim C., Lee Y., Kim H., Roh T., Yoo H.J. (2015). A multimodal stress monitoring system with canonical correlation analysis. 2015 37th Annual International Conference of the IEEE Engineering in Medicine and Biology Society (EMBC).

[130] Jeong D.H., Jeong J., Chae Y., Choi H.Y. (2017). Identification of attention state for menu-selection using in-ear EEG recording. 2017 5th International Winter Conference on Brain-Computer Interface (BCI).

